# Temperature downshifts induce biofilm formation in *Pseudomonas aeruginosa* through the SiaABCD signal and functional module

**DOI:** 10.1016/j.jbc.2025.111086

**Published:** 2025-12-22

**Authors:** Yanran Li, Zhe Chen, Tingying Xia, Yiqing Ding, Yingpeng Xie, Lu Miao, Zhaochao Xu, Xin Deng, Luyan Z. Ma, Aixin Yan

**Affiliations:** 1 School of Biological Sciences, The University of Hong Kong, Hong Kong SAR, China; 2 Department of Biomedical Sciences, City University of Hong Kong, Hong Kong SAR, China; 3 CAS Key Laboratory of Separation Science for Analytical Chemistry, Dalian Institute of Chemical Physics, Chinese Academy of Sciences, Dalian, China; 4 State Key Laboratory of Microbial Resources, Institute of Microbiology, Chinese Academy of Sciences, Beijing, China

**Keywords:** *Pseudomonas aeruginosa* (*P. aeruginosa*), biofilm, c-di-GMP metabolic enzymes, temperature response, cyclic di-GMP (c-di-GMP)

## Abstract

*Pseudomonas aeruginosa* is a highly adaptable Gram-negative pathogen known for its remarkable ability of forming biofilms. Understanding the environmental cues and regulatory mechanisms that drive biofilm formation is essential for developing effective control strategies. In this study, we screened 57 clinical and environmental *P. aeruginosa* isolates and discovered that a universal environmental cue, temperature downshift from host-associated 37 °C to room temperature (21 °C), significantly promotes biofilm formation in 63% of the strains. Using the ATCC 27853 strain as a model, we demonstrate that this enhancement results from increased production of the Psl exopolysaccharides at lower temperature. LC-MS/MS analysis revealed elevated levels of the secondary messenger c-di-GMP, a key regulator of the motile-to-sessile transition, at room temperature. Through screening a mutant library targeting 18 c-di-GMP metabolic enzymes, we identified the diguanylate cyclase SiaD within the SiaABCD signaling and functional module as a principal driver of c-di-GMP elevation and biofilm promotion. Further investigation showed that the entire SiaABCD module, especially the signal-sensing domain of SiaA, mediates the temperature-dependent response. Integrating lipidomics with genetics and physiological assays, we show that a temperature downshift triggers rapid membrane perturbations that activate the SiaABCD signaling module, thereby increasing Psl production to strengthen surface adhesion and drive robust biofilm formation. These findings establish temperature downshift as a previously unrecognized physiological cue that promotes biofilm formation in *P. aeruginosa* and define an adaptive regulatory pathway linking specific environmental stresses of membrane perturbation to dedicated c-di-GMP signaling module, paving the way for new strategies to disrupt biofilm-associated infections and transmission.

*Pseudomonas aeruginosa* is a prevalent and pernicious Gram-negative pathogen responsible for various infections. The species is notorious for its exceptionally large genome size (6.3 Mbp for the reference strain PAO1), numerous regulatory genes (approximately twice as many histidine kinase sensors as *Escherichia coli*), and a wide range of resistance determinants that confer remarkable adaptability, infectivity, and antibiotic resistance capabilities (1, 2, 3, 4). *P. aeruginosa* is also highly proficient at forming biofilms, which are matrix-encased multicellular aggregates of bacteria adhering to surfaces. Biofilm formation not only protects the pathogen from antibiotics and host immune attacks (5) but also promotes its survival in various environments and, consequently, its transmission. To manage biofilm formation and prevent pathogen transmission, it is crucial to identify environmental signals that trigger biofilm formation and understand the underlying regulatory mechanisms.

Temperature fluctuations are common environmental cues encountered by pathogens during infection and transmission cycles, such as those between room temperature (RT, 20–25 °C) and host-associated temperature (37 °C) (6). Living as poikilothermic organisms, bacteria are reported to be capable of sensing as little as 1 °C temperature fluctuations (7) and respond by modulating various processes (8, 9, 10). Several thermo-sensing machineries, such as RNA thermometers, protein thermometers, DNA topology/structure modules, and membrane fluidity (11), have been described to modulate the stress adaptations. In *P. aeruginosa*, several processes, including quorum sensing (QS) (6), type I, II, and III secretion systems (6, 12), phenazine biosynthesis (12), adhesion to polystyrene (13), and phage Pf1 expression (14), have been reported to be upregulated upon temperature upshifts. Conversely, CRISPR adaptation (15) and biofilm formation in certain strains (14, 16, 17) have been reported to be enhanced in response to temperature downshifts. However, the specific thermo-sensing mechanisms responsible for these physiological adaptations and the underlying regulatory mechanisms remain largely unknown. To date, only a handful of RNA thermometers and protein thermometers have been identified in *P. aeruginosa* (17, 18, 19, 20, 21), and they primarily respond to temperature upshifts. Stress adaptation to temperature downshifts, especially processes involved in *P. aeruginosa* infection and transmission such as biofilm formation, remains poorly understood.

A key factor driving the transition from motile single cells to biofilm formation is the secondary messenger cyclic di-guanosine 3′,5′-monophosphate (c-di-GMP) (22). It modulates biofilm development by binding to various effector proteins that control the expression of genes involved in biofilm formation or dispersal (22). Elevated c-di-GMP levels promote the transition from planktonic to sessile lifestyles and *vice versa* (23). The intracellular c-di-GMP level is controlled by the opposing activities of two classes of enzymes: diguanylate cyclases (DGCs) and phosphodiesterases (PDEs). DGCs, characterized by the GG[D/E]EF active site motif, synthesize c-di-GMP, whereas PDEs, containing EAL or HD-GYP domains, degrade it. Notably, the *P. aeruginosa* genome encodes one of the highest numbers of DGC and PDE genes among bacteria (23). Yet, these enzymes are not redundant and are proposed to modulate c-di-GMP levels at specific stages of the biofilm cycle and in response to distinct environmental and physiological signals (23). For example, the PDE DipA is activated in response to the dispersion-inducing nutrient glutamate through the NicD–BdlA–DipA pathway, leading to decreased c-di-GMP levels and promoting biofilm dispersal (24). Similarly, the PDE RmcA responds to light exposure, resulting in reduced c-di-GMP and delayed development of biofilm structures (25). However, for most DGCs and PDEs, the precise signals they detect and the specific stages of biofilm development they regulate remain unidentified.

In this study, we investigated biofilm formation in *P. aeruginosa* in response to the universal environmental cue of temperature fluctuations. We observed that a temperature downshift from the host-associated 37 °C to room temperature (RT, 21 °C) promoted biofilm formation in the majority of (>60%) clinical and environmental *P. aeruginosa* strain collection tested (total 57 strains). We discovered that this temperature downshift induced an elevation of c-di-GMP levels and promoted Psl polysaccharide production. Through screening a mutant library comprising deletions of each individual DGCs and PDEs in *P. aeruginosa* genome, we identified the DGC SiaD as the key regulator mediating this adaptive biofilm formation at RT. Furthermore, we demonstrated that the signal-perceiving protein SiaA in the SiaABCD signal transduction module plays a crucial role in regulating SiaD activity by sensing the physiological cue of membrane perturbations induced by temperature downshifts, which resemble the effects of detergent SDS treatment. These findings reveal an important patho-adaptation mechanism in *P. aeruginosa* and establish a framework for understanding how specific physiological stresses are detected and managed through dedicated modulation of c-di-GMP metabolism.

## Results

### Temperature downshifts enhanced biofilm formation in the majority of *P. aeruginosa* clinical and environmental isolates

**Figure 1:**
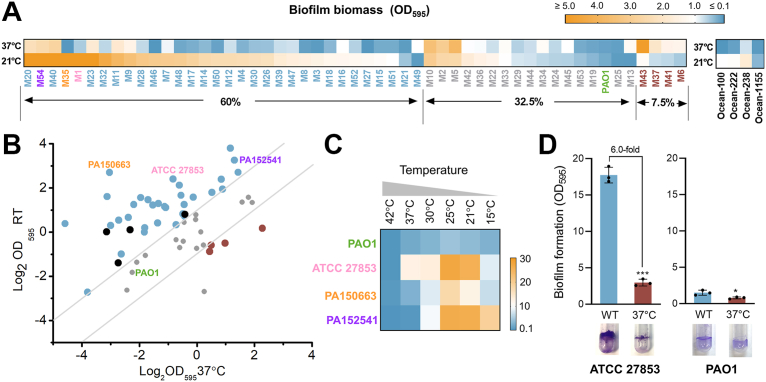
**Temperature downshifts enhanced biofilm formation in the majority of *Pseudomonas aeruginosa* clinical and environmental isolates.***A*, biofilm biomass of 52 clinical *P. aeruginosa* isolates, *P. aeruginosa* PAO1, and four environmental isolates following culturing statically at RT and 37 °C. M1: ATCC 27853. M35: PA150663. M54: PA152541. *B*, dot plot summarizing the biofilm patterns of *P. aeruginosa* isolates at RT and 37 °C. *Black*: environmental isolates. The two dash lines represent the threshold of 2-fold. *Blue*: strains formed two-fold or more biofilm at RT relative to 37 °C. *Red*: strains formed two-fold or more biofilm at 37 °C relative to RT. See related Figure S1 for bar graph with statistical analysis. *C*, biofilm biomass of ATCC 27853, PAO1, PA150663, and PA152541 at a temperature gradient from 15 °C to 42 °C at 48 h. *D*, biofilm formation of *P. aeruginosa* ATCC 27853 and PAO1 at RT and 37 °C. Data are represented as mean ± SD, n = 3 independent experiments. Asterisks indicate statistical significance. ∗, *p* < 0.05. ∗∗∗, *p* < 0.01 (based on Student’s *t* test).

To examine whether *P. aeruginosa* biofilm formation is affected by temperature fluctuations, particularly those between the hospital environment (RT, 21 °C) and the human host (37 °C), we measured the biofilm biomass of a collection of 52 clinical *P. aeruginosa* strains isolated from Queen Mary Hospital, Hong Kong and the reference strain PAO1 under the two temperatures (Figs. 1, *A* and *B*, and S1*A*). We found that 60% (32 out of 53) of the collection formed more than two-fold greater biofilm biomass at RT than at 37 °C (Fig.1, *A* and *B*). In contrast, only 7.5% (4 out of 53) of the collection formed more than two-fold biofilm biomass at 37 °C than at RT (Fig. 1, *A* and *B*). We further examined four environmental *P. aeruginosa* strains isolated from the surface layer of the North Pacific Ocean (26) and found that these strains also displayed higher biofilm biomass at RT than at 37 °C (Figs. 1, *A* and *B* and S1*B*). Monitoring biofilm formation in three representative strains, ATCC 27853, PA150663, and PA152541, which displayed a high degree of temperature responsiveness in the screening above (Fig. 1, *A* and *B*), across a broader temperature range (15–42 °C) confirmed that these strains exhibit a temperature-dependent biofilm formation pattern, with lower temperatures (21–25 °C) promoting biofilm formation and higher temperatures (>25 °C) inhibiting biofilm formation (Fig. 1*C*). Analyzing growth curve for the representative strain ATCC 27853 showed that its growth rate was similar to the reference strain (PAO1, Fig. S2), suggesting that the temperature-responsive biofilm formation pattern observed in ATCC 27853 is not attributed to differences in growth rates. These data indicate that temperature shifts represent a physiologically important signal that regulates biofilm formation in *P. aeruginosa*.

### Psl is primarily responsible for the enhanced biofilm formation at RT in *P. aeruginosa* ATCC 27853

**Figure 2:**
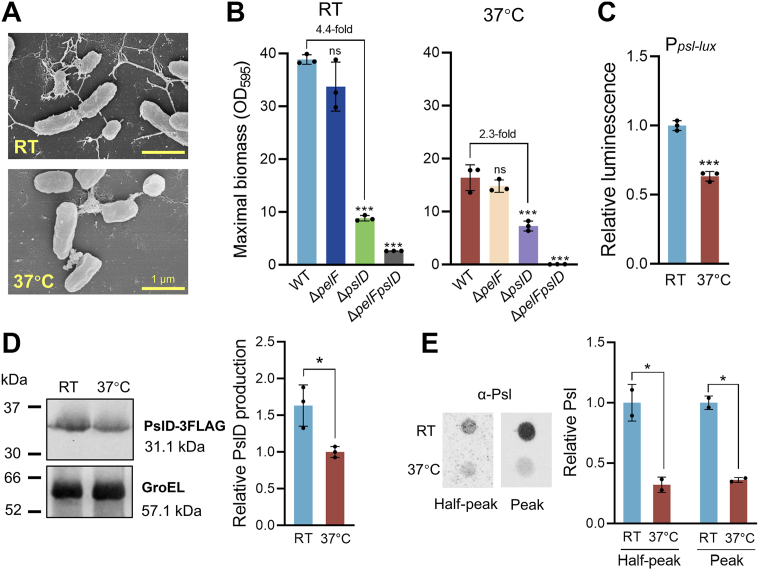
**Psl is primarily responsible for the enhanced biofilm formation at RT.***A*, SEM images of ATCC 27853 biofilm formed at RT (*upper*) and 37 °C (*lower*) on LB agar. Scale bar represents 1 μm. *B*, maximal biomass of ATCC 27853 and its isogenic Δ*pslD,* Δ*pslF*, and Δ*pslFpslD* mutants at RT and 37 °C. Data are represented as mean ± SD, n = 3 independent experiments. *C*, relative activity of the chromosomal P*psl*-*lux* transcriptional reporter in ATCC 27853 collected at half maximal biofilm at RT and 37 °C, respectively. Data are represented as mean ± SD, n = 3 independent experiments. *D*, Western blot of PslD-3FLAG in ATCC 27853 cultured at RT and 37 °C. Mid-log phase cells were collected for the analysis (*C–D*), that is, static cultures harvested at 24 h at RT and 8 h at 37 °C. Data are represented as mean ± SD, n = 3 independent experiments. *E*, relative Psl production of ATCC 27853 at RT and 37 °C as detected by immunoblot with Psl-specific antibody. *Left* panel, half peak biofilm time points (24h at RT and 8 h at 37 °C). *Right* panel, peak time points (48h at RT and 24 h at 37 °C). Data are represented as mean ± SD, n = 2 independent experiments. Asterisks indicate statistical significance. ∗, *p* < 0.05. ∗∗∗, *p* < 0.01. ns, not significant (based on Student’s *t* test). SEM, scanning electron microscopy.

Next, we employed ATCC 27853, a commonly studied *P. aeruginosa* strain with a complete genome sequence available (27, 28, 29, 30), to investigate adaptive biofilm formation in response to temperature downshift. This strain displayed both high biofilm formation capability and a high degree of temperature responsiveness among all strains tested (Figs. 1*D* and S1). Scanning electron microscopy analysis of ATCC 27853 biofilms formed at RT and 37 °C showed that, the biofilm formed at RT displayed an enhanced level of matrix production relative to 37 °C (Fig. 2*A*). It is well established that the *P. aeruginosa* biofilm matrix is mainly composed of three major extracellular polysaccharides: Psl, Pel, and alginate (31). To investigate which components contributes to the enhanced biofilm formation in response to the temperature downshifts, we constructed ATCC 27853 isogenic Δ*pslD*, Δ*pelF*, Δ*pslD*Δ*pelF,* and Δ*alg8* mutants and investigated their biofilm development over time. ATCC 27853 formed maximal biofilm at 48 h at RT and 24 h at 37 °C (Fig. S3). Deletion of *pslD* led to a significant reduction in biofilm biomass at RT, with a 4.4-fold decrease in maximal biomass (Fig. 2*B*). Notably, at 37 °C, Δ*pslD* also decreased biofilm formation, but to a much lesser extent, with a 2.3-fold reduction (Figs. 2*B* and S3). Deletion of *pelF* had minimal impact on biofilm formation at either temperature. The double deletion of both *pslD* and *pelF* completely abolished biofilm formation (Figs. 2*B* and S3). Deletion of *alg8*, which is involved in alginate biosynthesis (32), did not affect biofilm formation at either temperature (Fig. S4). These results suggest that both Psl and Pel constitute the biofilm matrix polysaccharides, with Psl being primarily responsible for the enhanced biofilm formation at RT.

We then constructed a chromosomal P*psl*-*lux* transcriptional fusion in ATCC 27853 to investigate the expression of *psl* genes in response to temperature fluctuations. The reporter activity peaked at 24 h at RT and 8 h at 37 °C, respectively (Fig. S5). As shown in Fig. 2*C*, the maximal activity of P*psl*-*lux* reporter was 1.6-fold higher at RT than at 37 °C. We also constructed a chromosomal *pslD*-3 × *flag* fusion in ATCC 27853 and observed that PslD protein levels were higher at RT than at 37 °C (Fig. 2*D*). Immunoblot analysis using Psl-specific antibody confirmed increased Psl production levels in biofilms formed at RT compared to 37 °C, at both half-peak and peak production time points (Fig. 2*E*). These results confirm that temperature downshift upregulates Psl production, thereby enhancing biofilm formation at RT in ATCC 27853.

### Enhanced Psl production at RT was mediated by the c-di-GMP–FleQ pathway in ATCC 27853

**Figure 3:**
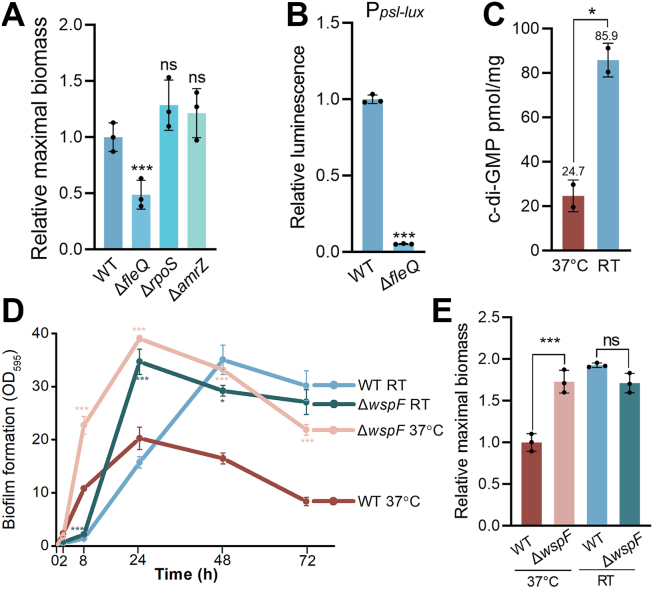
**Enhanced Psl production at RT was mediated by the c-di-GMP–FleQ pathway.***A*, relative maximal biofilm biomass of ATCC 27853 and its isogenic Δ*fleQ*, Δ*rpoS*, and Δ*amrZ* mutants at RT. Data are represented as mean ± SD, n = 3 independent experiments. *B*, relative activity of the chromosomal P*psl*-*lux* transcriptional fusion reporter collected at half maximal biofilm in ATCC 27853 and its isogenic Δ*fleQ* mutant at RT. Data are represented as mean ± SD, n = 3 independent experiments. *C*, intracellular c-di-GMP level at half maximal biofilm culture of ATCC 27853 at RT and 37 °C. Data are represented as mean ± SD, n = 2 independent experiments. *D*, dynamic biofilm formation of ATCC 27853 and its isogenic Δ*wspF* strain at RT and 37 °C. Data are represented as mean ± SD, n = 3 independent experiments. *E*, relative maximal biofilm biomass of ATCC 27853 and its isogenic Δ*wspF* strain at RT and 37 °C. Data are represented as mean ± SD, n = 3 independent experiments. Asterisks indicate statistical significance. ∗, *p* < 0.05. ∗∗∗, *p* < 0.01. ns, not significant (based on Student’s *t* test). c-di-GMP, cyclic di-guanosine 3′,5′-monophosphate.

Transcriptional factors known to regulate *psl* expression include the alternative σ-factor RpoS, the transcriptional repressor AmrZ, and a c-di-GMP effector FleQ (31). To investigate the regulation of *psl* expression at RT, we constructed isogenic Δ*rpoS*, Δ*amrZ*, and Δ*fleQ* mutants, respectively. While deletion of *rpoS* and *amrZ* had no effect on biofilm biomass, deletion of *fleQ* reduced biofilm formation (Fig. 3*A*). Deletion of *fleQ* also significantly reduced the P*psl*-*lux* activity in ATCC 27853 at RT (Fig. 3*B*), suggesting that FleQ plays an important role in regulating Psl biosynthesis and biofilm formation in ATCC 27853 at RT.

Since FleQ acts as a c-di-GMP effector, we performed LC-MS/MS analysis and found that ATCC 27853 cells cultured at RT displayed 3.5-fold higher c-di-GMP levels than in cells cultured at 37 °C (Fig. 3*C*). These results suggest that temperature downshift induced an elevation of c-di-GMP levels, which promotes biofilm formation by enhancing FleQ-regulated Psl production. To confirm the c-di-GMP–dependent mechanism of biofilm promotion, we constructed an isogenic Δ*wspF* mutant, which constitutively produces high levels of c-di-GMP (33, 34), and examined its biofilm formation at the two temperatures (Fig. 3, *D* and *E*). As expected, at 37 °C, the Δ*wspF* mutant formed significantly more biofilm than the WT (Fig. 3, *D* and *E*). At RT, however, Δ*wspF* did not further increase the maximal biofilm biomass (Fig. 3, *D* and *E*), suggesting that c-di-GMP–driven biofilm output in ATCC 27853 is already near saturation at RT. Notably, biofilm development in Δ*wspF* was accelerated at RT (Fig. 3*D*), with maximal biomass peaking at 24 h instead of 48 h in the WT. These results indicate that in the ATCC 27853 WT, temperature downshift induces an adaptive rise in c-di-GMP, resulting in an enhanced but delayed peak in biofilm biomass at RT relative to 37 °C. Collectively, these results confirm that the increased biofilm formation observed at RT in ATCC 27853 is primarily driven by elevated c-di-GMP.

### Temperature downshift induces biofilm formation in ATCC 27853 through the SiaABCD signaling module

**Figure 4:**
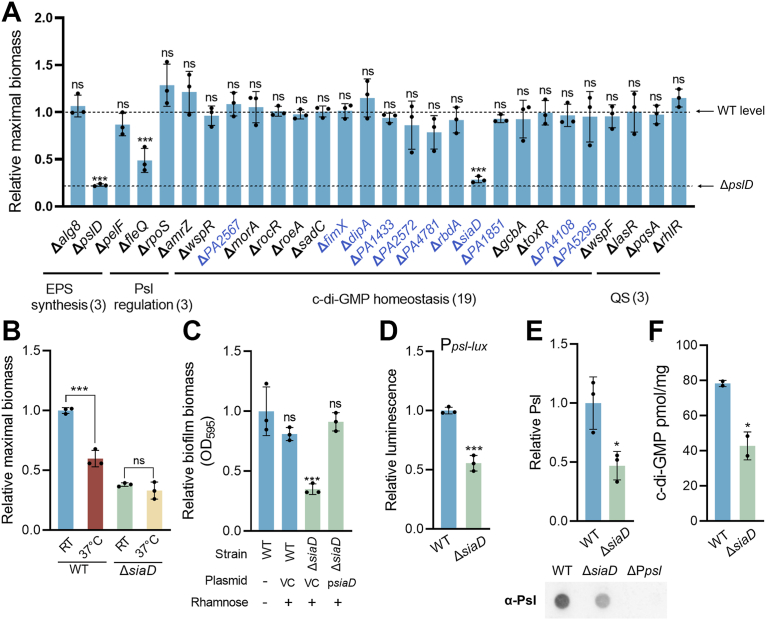
**The DGC SiaDisprimarily responsible for the elevated c-di-GMP content and enhanced Psl production at RT in ATCC 27853.***A*, relative maximal biofilm biomass of 28 isogenic mutants relative to the ATCC 27853 WT at RT. *Blue*, genes identified in RNA-seq analysis. *B*, relative maximal biofilm formation of ATCC 27853 and its isogenic Δ*siaD* strain at RT and 37 °C. *C*, relative maximal biofilm formation of ATCC 27853, isogenic Δ*siaD* mutant, and Δ*siaD* mutant complemented with rhamnose-inducible expression of *siaD* at 48 h at RT. VC, the pJM253 vector control. *D*–*F*, relative activity of the chromosomal P*psl*-*lux* transcriptional fusion reporter (*D*), Psl level in biofilm matrix (*E*), and intracellular c-di-GMP level (*F*) in ATCC 27853 and its isogenic Δ*siaD* mutant at RT. Midlog phase static cells harvested at 24 h at RT were collected for the analysis in (*C–F*). Data are represented as mean ± SD. (A–E), n = 3 independent experiments. (*F*), n = 2 independent experiments. Asterisks indicate statistical significance. ∗, *p* < 0.05. ∗∗∗, *p* < 0.01. ns, not significant (based on Student’s *t* test). c-di-GMP, cyclic di-guanosine 3′,5′-monophosphate; DGC, diguanylate cyclase.

To examine the molecular mechanisms underlying this response, we performed a RNA-seq analysis to comprehensively profile gene expression in biofilm cells at RT and 37 °C. It was found that a large number of genes involved in c-di-GMP metabolism, including both DGCs and PDEs, were differentially expressed between the two temperatures (Table S4). To identify which DGCs or PDEs are responsible for the elevated c-di-GMP and enhanced biofilm formation at RT, we generated gene deletion mutants for 18 genes encoding these enzymes (Table S5). These included genes that showed differential expression in the RNA-seq analysis as well as those previously reported to regulate biofilm development (23). Examining biofilm formation in these mutants at RT and 37 °C revealed that deletion of the DGC gene *siaD* resulted in a significant reduction in biofilm formation at RT (Figs. 4*A* and S6), comparable to the reduction observed in the Δ*pslD* mutant.

Furthermore, deletion of *siaD* resulted in a markedly reduced temperature responsiveness of biofilm formation compared to the WT (Fig. 4*B*), further suggesting that SiaD plays a major role in mediating the biofilm enhancement triggered by temperature downshift. Complementation experiments confirmed the role of *siaD* in promoting biofilm formation at RT (Fig. 4*C*). The Δ*siaD* mutant also showed decreased P*psl*-*lux* reporter activity and reduced Psl levels in the biofilm matrix of ATCC 27853 at RT (Fig. 4, *D* and *E*). LC-MS/MS revealed a reduction in c-di-GMP levels in ATCC 27853 Δ*siaD* cells compared to the WT (Fig. 4*F*). Collectively, these results strongly suggest that the activation of the DGC *siaD* is responsible for the elevated c-di-GMP levels and enhanced biofilm formation in response to temperature downshift.

**Figure 5:**
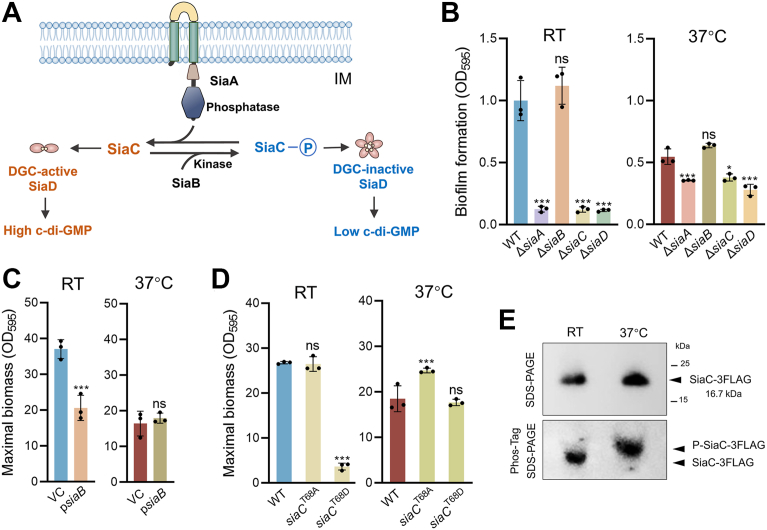
**Temperature downshift from 37 °C to RT induces biofilm formation in ATCC 27853 through the SiaABCD signaling module.***A*, schematic diagram of the SiaABCD signaling and functional pathway. *B*, relative maximal biomass of ATCC 27853 and its isogenic Δ*siaA*, Δ*siaB,* Δ*siaC,* and Δ*siaD* mutants at RT and 37 °C. Data are represented as mean ± SD, n = 3 independent experiments. *C*, maximal biomass of ATCC 27853 supplemented with a vector control (VC) or rhamnose-inducible *siaB* at RT and 37 °C. Data are represented as mean ± SD, n = 3 independent experiments. *D*, maximal biomass of ATCC 27853 and its isogenic mutants carrying *siaC* alleles encoding a phosphorylation-deficient (SiaC^T68A^) or phosphomimetic (SiaC^T68D^) mutant at RT and 37 °C. Data are represented as mean ± SD, n = 3 independent experiments. *E*, phosphorylation status of SiaC-3FLAG at RT and 37 °C detected by Phos-tag SDS-PAGE. Asterisks indicate statistical significance. ∗, *p* < 0.05. ∗∗∗, *p* < 0.01. ns, not significant (based on Student’s *t* test).

SiaD encodes a GGEEF domain–containing DGC that was initially reported to regulate macroscopic cell aggregation in the presence of the surfactant SDS in *P. aeruginosa* (35, 36). Recently, *siaABCD* was reported to be cotranscribed as an operon, and the encoded proteins are involved in regulating aggregation and biofilm formation in PAO1 (37, 38, 39). In the SiaABCD signal transduction module, the inner membrane protein SiaA acts as a Ser/Thr phosphatase to dephosphorylate SiaC, and SiaB works antagonistically as a kinase to phosphorylate SiaC (Fig. 5*A*). The reversible phosphorylation of SiaC acts as a “switch” to modulate the activity of SiaD *via* direct interaction, where unphosphorylated SiaC induces the DGC activity of SiaD and phosphorylated SiaC inhibits the DGC activity of SiaD (37, 38, 39) (Fig. 5*A*).

To investigate whether the entire SiaABCD module is involved in sensing temperature downshift and enhancing biofilm formation, we constructed isogenic mutants Δ*siaA* and Δ*siaC*, which are expected to exhibit effects similar to the Δ*siaD* mutant on biofilm formation, and a Δ*siaB* mutant, which should display an opposite effect compared to Δ*siaA*, Δ*siaC*, and Δ*siaD*. As anticipated, deletion of *siaA* or *siaC* resulted in a dramatic reduction in biofilm formation at RT, evidenced by decreased maximal biofilm biomass, similar to that observed in Δ*siaD* (Fig. 5*B*). Conversely, deletion of *siaB* had a negligible effect on biofilm formation (Fig. 5*B*), but overexpression of *siaB* through a rhamnose-inducible system reduced biofilm formation at RT (Fig. 5*C*). Together, these results confirm the regulatory role of the entire SiaABCD signal transduction module in mediating enhanced biofilm formation in ATCC 27853 at RT.

To further validate this, we constructed mutants expressing a phosphomimetic SiaC variant (SiaC^T68D^), which is expected to result in constitutively low DGC activity of SiaD, and a phosphorylation-deficient SiaC variant (SiaC^T68A^), which should lead to sustained high DGC activity. Biofilm assays revealed that introduction of the phosphomimetic variant SiaC^T68D^ significantly abolished biofilm formation at RT, whereas the SiaC^T68A^ variant formed comparable biofilm to the WT strain at RT (Fig. 5*D*). These results suggest that at RT, SiaC primarily exists in its unphosphorylated form, promoting the DGC SiaD and biofilm formation. We also examined the effect of *sia* deletion mutants and *siaC* variants on biofilm formation at 37 °C. Although Δ*siaA*, Δ*siaC*, and Δ*siaD* also led to slight reductions in biofilm formation (Fig. 5*B*), introduction of the phosphomimetic SiaC^T68D^ did not alter biofilm formation (Fig. 5*D*), while the phosphorylation-deficient SiaC^T68A^ increased biofilm formation (Fig. 5*D*). These findings indicate that SiaC primarily exists in its phosphorylated form at 37 °C and the SiaABCD module contributes minimally to biofilm regulation under this condition. Phos-tag SDS-PAGE confirmed these phosphorylation states of SiaC under the two temperatures (Fig. 5*E*). Furthermore, Δ*siaA* also led to reduced Psl production and decreased biofilm formation in another clinical strain, PA150663, at RT (Fig. S7), suggesting that this SiaABCD-mediated response to temperature downshift is conserved across different strains.

### SiaA senses temperature downshift–induced membrane perturbations to activate SiaABCD signaling module

**Figure 6:**
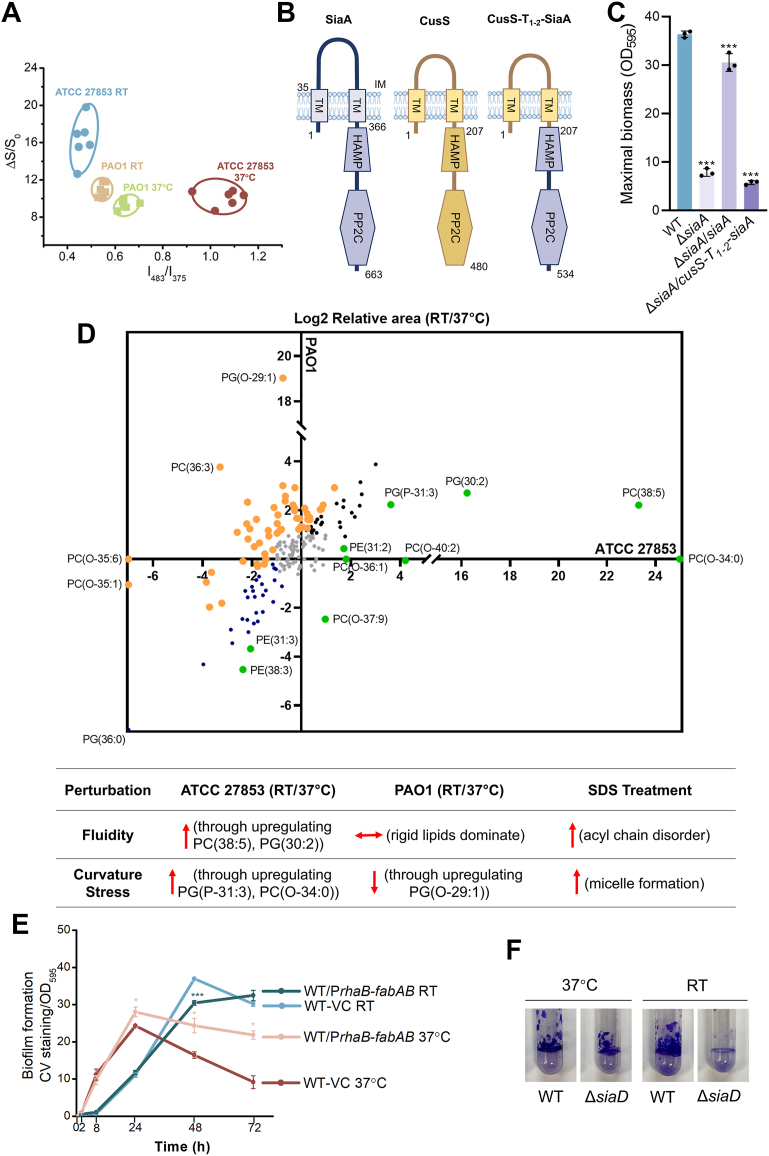
**Temperature downshifts induce distinct membrane perturbations to activate the SiaABCD signaling module.***A*, 2D graph plotted from output signals of two channels of the PI-BactD probe, fluorescence increase (ΔS/S_0_), and fluorescence radiometric changes (I_483_/I_375_), in ATCC 27853 and PAO1 at RT and 37 °C. Each assay was conducted in six replicates. *B*, schematic diagram showing the domain structures of WT SiaA, WT CusS, and CusS-SiaA chimeric variant. *C*, maximal biomass of ATCC 27853 WT strain, Δ*siaA* mutant, and Δ*siaA* mutant carrying chromosomally integrated CusS-SiaA chimeric variant at RT. Data are represented as mean ± SD, n = 3 independent experiments. *D*, *upper* panel, Log2 relative area of membrane lipid groups of ATCC 27853 and PAO1 as detected by lipidomics analysis (RT/37 °C). *Orange* and *green*: differentially changed in ATCC 27853 relative to PAO1 in response to temperature shift. *Black* and *blue*: synchronously changed in ATCC 27853 and PAO1 in response to temperature shift. *Gray*: similar levels at RT and 37 °C. *Lower* panel, a table summarizing the membrane perturbations upon temperature downshift and SDS treatment. *E*, dynamic biofilm formation of ATCC 27853 harboring either a vector control (VC) or rhamnose-inducible *fabAB* construct at RT and 37 °C. VC, the pJM253 vector control. Data are represented as mean ± SD, n = 3 independent experiments. *F*, pellicle formation of ATCC 27853 at RT and 37 °C collected at half-maximal biofilm stage, that is, 24h at RT and 8h at 37 °C, OD_600_ ∼0.4. Asterisks indicate statistical significance. ∗, *p* < 0.05. ∗∗∗, *p* < 0.01 (based on Student’s *t* test).

The Sia (SDS-induced aggregation) system was initially identified as a regulator of *P. aeruginosa* cell aggregation in response to the membrane-disrupting agent SDS (35, 36). SDS has been reported to induce membrane stress in the form of spontaneous curvature, arising from its rapid insertion into the outer leaflet and slower translocation to the inner leaflet of the bacterial lipid bilayer (40). Notably, membrane stress, such as changes in fluidity, is recognized as a key bacterial temperature sensor (11). Based on these notions, we hypothesized that SiaA may sense membrane perturbations caused by temperature downshift in ATCC 27853, thereby activating the SiaABCD signal transduction module. To test this, we first employed a membrane-sensitive probe PI-BactD (41) to monitor the physicochemical properties of ATCC 27853 at RT and 37 °C (Figs. 6*A* and S8). The probe reports membrane surface properties *via* two orthogonal readouts: the overall fluorescence increase reflecting probe disassembly upon binding to the anionic bacterial surface and the pyrene excimer-to-monomer emission ratio (I_482_/I_375_) capturing differences in local surface interactions and thus membrane surface properties. While the 2D output signals from PAO1 membranes at RT and 37 °C clustered closely together, ATCC 27853 exhibited clear separation between temperatures (Fig. 6*A*). This distinct clustering indicates that ATCC 27853 undergoes more substantial membrane perturbation upon temperature downshift, acting as a plausible signal to activate the SiaABCD module.

To further investigate this mechanism, we focused on the signal-sensing domain of SiaA, which resembles the transmembrane sensor kinase component of the two-component system in bacteria. These proteins typically possess an N-terminal signal perception transmembrane domain and a C-terminal cytoplasmic effector domain involved in phosphate transfer (36, 38). We engineered a chimeric SiaA variant in which the first 336 amino acids, encompassing the signal-sensing domain, were replaced with the corresponding regions from the well-studied sensor kinase protein *E. coli* CusS (42), resulting in the chimeric CusS-T_1-2_-SiaA protein (Fig. 6*B*). Biofilm assays demonstrated that while native SiaA sustained a high level of biofilm at RT, the chimeric construct CusS-T_1-2_-SiaA failed to do so (Fig. 6*C*). Western blot analysis confirmed comparable expression levels of CusS-T_1-2_-SiaA and the WT SiaA (Fig. S9). These results suggest that the signal-sensing domain of SiaA plays an important role in regulating temperature downshift–induced biofilm formation in ATCC 27853.

Next, we performed lipidomics analysis to compare the lipid compositions of ATCC 27853 and PAO1 biofilm cells at RT and 37 °C. The analysis revealed significant, strain-specific changes in membrane lipids upon temperature downshift in ATCC 27853, with 35.3% of the lipid groups displaying distinct responses compared to PAO1 (Fig. 6*D*, upper panel). Specifically, ATCC 27853 exhibited marked upregulation of fluidity-promoting unsaturated lipids PC(38:5) and PG(30:2) and curvature-inducing lipids PG(P-31:3), along with unique production of plasmalogen-like lipid PC(O-34:0) and depletion of hyperfluid lipids such as PC(O-35:6). These changes collectively produced a membrane state resembling SDS-treated cells — increased fluidity with pronounced curvature stress (Fig. 6*D*, lower panel). Conversely, PAO1 maintained membrane homeostasis by upregulating rigid lipids such as PG(O-29:1) and PC(36:3) at RT (Fig. 6*D*), thereby avoiding such stress. This fundamental difference in membrane adaptation strategies likely underpins the unique capacity of ATCC 27853 to transduce temperature downshifts into c-di-GMP–mediated biofilm induction *via* the SiaABCD signaling pathway, while PAO1 remains insensitive.

Consistent with this observation, our RNA-seq analysis of ATCC 27853 also revealed differential expression in genes involved in fatty acids biosynthesis and degradation following a temperature downshift from 37 °C to RT (Table S6). Specifically, compared with biofilms harvested at 37 °C, those collected at RT exhibited a coordinated upregulation of key fatty acids biosynthesis genes, including *fabF1*, *fabG*, *fabZ*, *fabD*, and *accC*. The upregulation of *fabZ*, which encodes a β-hydroxyacyl-ACP dehydratase, is particularly notable as it functions to introduce the double bonds characteristic of unsaturated fatty acids. Furthermore, RNA-seq analysis also revealed a strong upregulation of the fatty acid-CoA ligase gene *fadD1*, which is involved in the activation and degradation of long-chain fatty acids, concurrent with a downregulation of its paralog *fadD2*, which preferentially processes shorter-chain fatty acids relative to the FadD1 enzyme. This pattern suggests a shift in substrate preference and signifies that the membrane adaptation involves not only *de novo* synthesis of unsaturated lipids but also active remodeling of the existing membrane lipid pool. These transcriptional changes are fully consistent with the observed lipidomic shift towards shorter, more unsaturated fatty acid chains that enhance membrane fluidity at lower temperatures.

The cytoplasmic membrane is among the first cellular components to experience temperature shifts. A sudden downshift can immediately alter membrane physical properties—such as fluidity, curvature, and lateral pressure profiles—before compositional homeoviscous adaptation can occur (43). To distinguish whether SiaA senses spontaneous membrane perturbations directly or responds to membrane composition changes induced by the temperature downshift, we genetically manipulate membrane composition independently of temperature by introducing *fabA* and *fabB*—key genes for unsaturated fatty acid synthesis—under a rhamnose-inducible promoter in ATCC 27853. Analysis of biofilm dynamics revealed that at RT, where the membrane is already physically perturbed by the downshift, *fabAB* induction did not further increase biofilm formation across the 72-h measurement (Fig. 6*E*), suggesting that altered lipid composition *per se* is not the immediate trigger for SiaA-dependent biofilm induction. At 37 °C, *fabAB* induction enhanced biofilm formation, but only at later time points (after 8 h), with no detectable effect during the early stage (0–8 h) (Fig. 6*E*). Thus, increasing unsaturated fatty acid pool and membrane fluidity can promote biofilm formation, but its impact is delayed, reflecting a downstream amplifying role rather than an initial cue. Together, these data support a model in which temperature downshift elicits immediate physical perturbations in the membrane, which are directly detected by SiaA through its sensory domain, activating the SiaABCD module. This early response, alongside other cold-shock pathways, then drives transcriptional changes—including the upregulation of *fab* genes—that remodel the lipidome to adapt to the new temperature. This adaptive remodeling helps preserve membrane function at low temperature and may feedback on SiaA signaling to sustain elevated c-di-GMP and the biofilm state.

To examine the role of SiaABCD signaling during early biofilm development, we assessed surface adherence of ATCC 27853. ATCC 27853 exhibited markedly stronger surface attachment at RT than at 37 °C, as evidenced by pellicle formation in log phase cells (OD_600_ of 0.4, 24 h for cells cultured at RT and 8 h for cells cultured at 37 °C) (Fig. 6*F*). This attachment was significantly reduced in the Δ*siaD* mutant, consistent with its diminished Psl production (Fig. 4*E*). Collectively, these results indicate that temperature-induced membrane perturbations activate the SiaABCD pathway, thereby promoting biofilm formation through both Psl-mediated early-stage adhesion and matrix production during overall biofilm development.

## Discussion

**Figure 7:**
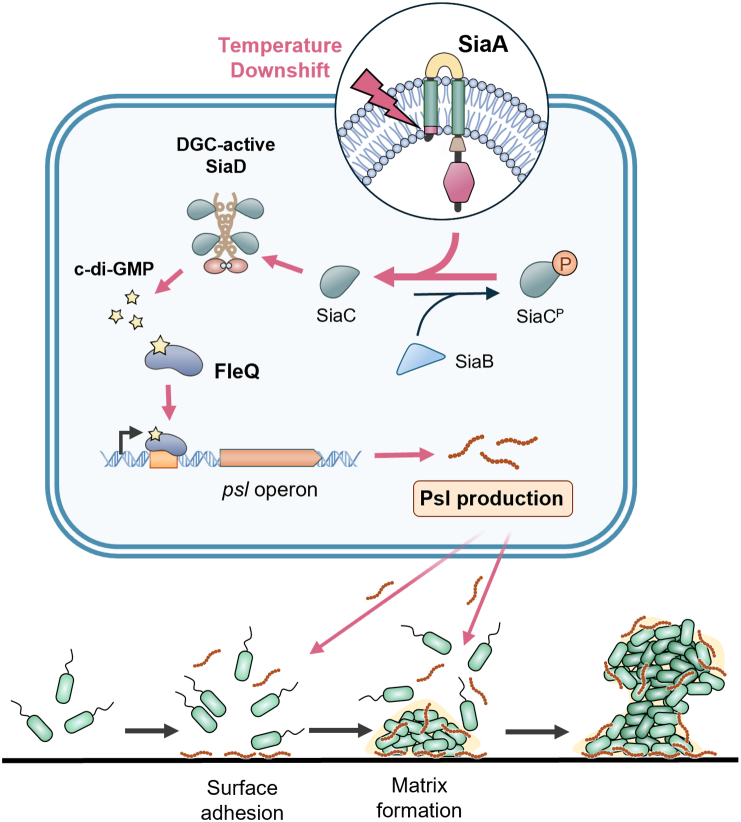
**A model to illustrate the activation of SiaABCD and its mediated biofilm enhancement in ATCC 27853 in response to temperature downshifts.** Temperature downshift from 37 °C to RT causes membrane perturbations in ATCC 27853, which induce SiaA phosphatase activity to promote the formation of SiaC–SiaD complex and DGC activity of SiaD. The activated Sia system regulates *psl* production *via* c-di-GMP–FleQ–Psl pathway. Increased Psl production enhanced biofilm formation of ATCC 27853 at RT through enhancing both surface attachment and biofilm matrix development. c-di-GMP, cyclic di-guanosine 3′,5′-monophosphate; DGC, diguanylate cyclase.

Temperature fluctuations are common environmental cues encountered by bacterial pathogens during their transition from environmental reservoirs to mammalian hosts. Several studies have shown that pathogens rewire their gene expression to upregulate virulence factors and downregulate survival determinants in response to temperature upshifts from RT to host-associated temperatures (12). Recently, several clinical *P. aeruginosa* strains (J16, J108, J153, J161, and J202) isolated from Pusan Paik Hospital, Busan, South Korea (16, 17, 44) were reported to exhibit enhanced biofilm formation in response to temperature downshifts. However, the underlying regulatory mechanisms were unknown. In this study, we showed that temperature downshift is a common cue that enhances biofilm formation in 63% (36 out of 57) of a collection of clinical and environmental *P. aeruginosa* strains tested. We showed that the exopolysaccharide Psl was primarily responsible for the enhanced biofilm formation at RT compared to 37 °C in the model temperature-responsive strain ATCC 27853. Through screening an extensive library of gene deletions encoding DGCs or PDEs, we identified SiaD, the DGC within the SiaABCD signaling module, as the key regulator responsible for increased Psl level *via* elevated c-di-GMP production. Further investigation revealed that the entire SiaABCD module is required for this regulation, with the sensor domain of the membrane protein SiaA playing a crucial role in signal detection. Lipidomics, combined with various genetic and physiological assays, indicated that temperature downshift–induced membrane perturbations in ATCC 27853 activate SiaABCD, leading to increased Psl production. This, in turn, enhances surface attachment and matrix formation, promoting robust biofilm development. A working model summarizing these findings is illustrated in Figure 7.

Signal transduction systems are crucial for bacterial survival in fluctuating environments, as they regulate cellular responses to adapt to changing conditions (45). The bacterial c-di-GMP signaling module typically consists of four components: two enzymes that produce and degrade the signaling molecule, an effector, and a target component with functional output (22). Compared to other classic secondary messenger control modules, such as the cAMP signaling system, where a single adenylate cyclase or a specific PDE synthesizes or degrades the signaling molecule (cAMP) and binds to a single transcriptional factor effector CRP (cAMP-receptor protein) (46), the c-di-GMP signaling module exhibits extraordinary multiplicity in each of its four principal components. Uncovering the full spectrum of the c-di-GMP signaling network requires identifying all members involved in each of the four principal components and their specific functions. Gram-negative bacteria, especially Gammaproteobacteria, display a striking proliferation of GGDEF and EAL domain proteins. Among them, *P. aeruginosa* encodes one of the highest numbers of DGCs and PDEs in its genome, which are proposed to respond to diverse signals the bacterium encounters in its habitats and have distinctive impacts on bacterial phenotype (47, 48). Most DGCs and PDEs contain an N-terminal sensory input domain that integrates diverse environmental and cellular signals into the c-di-GMP signaling network by modulating the corresponding DGC or PDE activities. Identified signals perceived by these sensory domains include oxygen and redox conditions, light, starvation, and various extracellular substances, such as antibiotics, polyamines, or intracellular signaling molecules (49).

Unlike these DGCs and PDEs, which directly sense stimuli through their N-terminal sensory domains, the activity of the DGC SiaD is regulated in a partner-switch system fashion (37, 38, 39). In this model, SiaD is activated by the binding of unphosphorylated partner SiaC, and the phosphorylation status of SiaC is controlled by the kinase protein SiaB and the phosphatase SiaA, a membrane protein with two transmembrane domains. Previously, the SiaABCD module was reported to sense effects induced by exogenous toxic compound TeO_3_^2−^ and the detergent SDS, thereby promoting biofilm formation in *P. aeruginosa*. In this study, we revealed that the physiological signal detected by the SiaABCD module is membrane perturbations induced by temperature downshifts. A well-studied sensor kinase that serves as a paradigmatic transmembrane thermosensor is the *Bacillus subtilis* DesK protein, which senses membrane viscosity changes upon temperature downshifts (below 30 °C) and regulates lipid desaturation to counteract the perturbation (10, 50, 51). A “sunken-buoy” (SB) motif in the TM1 of DesK encompassing three hydrophilic amino acids (Q9, K10, and N12), which are unusual in TM sequences (52), were found to be important for perceiving temperature downshift. We examined the TM sequences of SiaA and identified three polar amino acids, R8, K10, and S11, that are similar to the SB motif in DesK and represent a potential mechanism for temperature sensing by SiaA (Figs. S10 and S11). The lipidomic signature of ATCC 27853 characterized by fluidity-enhancing and curvature-inducing lipids provided a physicochemical mechanism for the SiaA-mediated signal sensing. Collectively, these studies suggest that the SiaABCD module may act as a primary signal transduction system that senses membrane perturbations in *P. aeruginosa* under various stress conditions and elicits the functional output of biofilm formation through c-di-GMP signaling.

Although the SiaABCD signaling module is conserved across *P. aeruginosa*, some strains, including PAO1, do not exhibit temperature-responsive biofilm formation. Our lipidomic and physiological analyses reveal strain-specific membrane properties and adaptive responses that likely underlie these divergent temperature-dependent biofilm phenotypes. To comprehensively identify determinants of the differential response, we pursued comparative genomics. The analysis, however, was challenged by the species' complex genome architecture—comprising a conserved core and a highly variable accessory component—and by the fact that approximately 45% of PAO1 genes have unknown function (53). Guided by these genomic insights, we pursued specific leads. For instance, the accessory genome of ATCC 27853 is enriched in prophages and genomic islands and notably lacks the *wbp* gene cluster responsible for B-band LPS O-antigen biosynthesis present in PAO1 (30). Since this loss could alter surface hydrophobicity, we tested its potential contribution to temperature responsive biofilm formation by constructing a PAO1 Δ*wbpM* mutant. The mutant displayed increased biofilm formation at both temperatures but remained temperature-nonresponsive, mirroring the PAO1 parent (Fig. S12). This indicates that while certain surface composition modulates the magnitude of biofilm formation, this difference alone does not account for temperature responsiveness. Overall, our findings suggest that strain differences in temperature responsiveness are unlikely to stem from the mere presence or absence of core components (SiaABCD, FleQ, Psl); instead, they likely reflect nuanced variation in regulation or activity, potentially involving genes of unknown function. Systematic comparative genomics integrated with targeted functional assays across a broader strain panel will be required to pinpoint the specific genetic determinants.

Furthermore, as a minor methodological consideration, future studies should quantify gene expression and other assays in rigorously separated planktonic subpopulations alongside biofilm cells. In our current setup, biofilms form at the air–liquid interface and on vessel walls, while the broth contains free-living cells plus a small fraction of dispersed biofilm fragments, complicating the recovery of uncontaminated planktonic samples for sensitive assays. Implementing dedicated fractionation strategies, such as flow-cell or reactor platforms with defined sampling ports and gentle biofilm harvesting, should enable subpopulation-resolved measurements and refine our understanding of the regulatory dynamics underlying temperature downshift–induced biofilm formation. Moreover, a technical consideration for the *luxCDABE* reporter system we employed is its reliance on endogenous FMNH_2_ availability. Cellular FMNH_2_ levels are influenced by metabolic state, which can vary with environmental factors such as growth temperature, potentially affecting the bioluminescent signal independently of promoter activity. While our P*psl*-*lux* activity measurements at 0-6h (Fig. S5) showed comparable signals at RT and 37 °C, suggesting that FMNH_2_ was not differentially limiting during this early biofilm stage, this does not fully exclude its influence under later stages of biofilm development. Implementing an internal control, such as a strong, constitutive *lux* reporter, would allow for normalization against FMNH_2_ pool fluctuations, thereby strengthening future quantitative analyses. Lastly, while our study characterized the initial stages of biofilm development triggered by a temperature downshift, we have not yet examined the effects of a temperature upshift on established biofilms. Determining whether a return to host temperature serves as a cue for biofilm dispersal is a compelling direction for future research, as it mirrors the transition bacteria undergo from environmental reservoirs to the human host.

Nonetheless, biofilm formation represents an important strategy employed by bacterial pathogens for adaptation and persistence in fluctuating environments. Our findings that the majority of *P. aeruginosa* isolates display enhanced biofilm formation in response to temperature downshifts from host-relevant 37 °C to RT, and that the SiaABCD regulatory module plays an important role in this process, may lead to targeted anti-biofilm and anti-infection strategies. Given the prominent role of SiaA and its broad distribution and conservation across bacterial taxa, blocking SiaA-mediated signal transduction may reduce biofilm formation and transmission of *P. aeruginosa* in environment-host transmission cycles, thereby contributing to the control of this pathogen.

## Experimental procedures

### Bacterial strains

Bacterial strains used and constructed in this study are listed in Table S1. *E. coli* DH5a was used for plasmid propagation. *E. coli* SM10*λpir* was used for conjugal transfer of plasmids. *P. aeruginosa* clinical strains were isolated from Queen Mary Hospital, Hong Kong. *P. aeruginosa* environmental strains were isolated from the surface layer of the North Pacific Ocean (26). *P. aeruginosa* mutants were generated as described in Method details. Generally, *P. aeruginosa* and *E. coli* strains were grown in lysogeny broth (LB). When necessary, 100 μg/ml ampicillin, 10 μg/ml tetracycline, or 25 μg/ml chloramphenicol was supplemented in *E. coli* culture and 300 μg/ml carbenicillin or 100 μg/ml tetracycline was supplemented in *P. aeruginosa* culture.

### Biofilm assay

Biofilm formation of *P. aeruginosa* was quantified following a previous description with slight modifications (54). For biofilm screening in 96-well plate, overnight culture of *P. aeruginosa* was diluted to OD_600_ as 0.1 with fresh LB medium, and 100 μl of the subculture was transferred to each well of a clear flat-bottom polystyrene 96-well plate (Corning). For dynamic biofilm formation of individual strains, overnight culture of *P. aeruginosa* was diluted to OD_600_ as 0.1 with M63 medium (KH_2_PO_4_ 13.6 g/L, (NH_4_)_2_SO_4_ 2.0 g/L, FeSO_4_ 0.5 mg/L, MgSO_4_ 0.2 g/L, casamino acids 5.0 g/L, glucose 2.0 g/L, arginine 4.0 g/L) and 0.5 ml of the subculture was transferred to a 5-ml round-bottom polypropylene tube. After incubating statically, the culture medium was removed, and the microtiter plate was gently washed three times with water. Biofilm cells were stained with 0.1% crystal violet (200 μl for 96-well plate culture, 1 ml for tube culture) for 10 min, washed three times with water, and air dried. For both 96-well plate culture and tube culture, 125 μl 30% acetic acid was added to dissolve biofilm and 100 μl of the solubilized suspension was transferred to a microtiter plate. The absorbance at 595 nm was measured with a Synergy HTX Multi-Mode Microplate Reader (BioTek).

### Scanning electron microscopy

Overnight culture of ATCC 27853 was diluted as OD_600_ as 0.1 with fresh LB medium, and the subculture was spread onto 12 mm cover glass on LB agar plates. After incubating for 48 h, 0.4% formalin in 0.1 M phosphate saline buffer (pH 7.4) was added on the cover glass, and the biofilm cells were fixed at 4 °C overnight. The cover glass was then washed with PBS buffer and treated with an upgrading series of ethanol. The dehydrated samples were subjected to critical point drying. Following that, the samples were sputtered coat with Au/Pd coating to enhance the conductivity. The samples were then examined by scanning electron microscopy facility in HKU (Hitachi S4800 FEG SEM, Hitachi Ltd).

### Promoter-*lux* fusion and its integration into *P. aeruginosa* genome

A 350 bp DNA fragment corresponding to the promoter region of *pslA* was amplified and cloned to the vector pmini-CTX-*lux* (55) at the HindIII site. The reporter was integrated to the *attB* site of *P. aeruginosa* cells. To examine reporter activity, overnight culture of P*psl*-*lux* reporter-containing cells in M63 medium was diluted to OD_600_ as 0.1 in a 14 ml Falcon tube with a total volume of 3 ml. Cell cultures were incubated statically at RT or 37 °C. At each time point, 200 μl of the cultures were transferred to a 96-well plate to measure the luminescence intensity and OD_600_ with a Synergy HTX Multi-Mode Microplate Reader (BioTek) at ambient temperature.

### Quantification of intracellular c-di-GMP level

Quantification of c-di-GMP level was conducted based on the method described previously with slight modifications (56). Overnight culture of *P. aeruginosa* cells in M63 medium was diluted to OD_600_ as 0.1 and incubated statically at RT or 37 °C for 24 h. Three milliliters bacterial culture was pelleted at 4 °C, resuspended in 990 μl M63 medium, and 10 μl 1 μM 2-chloro-AMP was added. 94.2 μl 70% v/v perchloric acid (HClO_4_) was added to lyse cells, and the samples were vortexed and immediately incubated on ice for 30 min. After centrifuging at 13,000 rpm, 4 °C for 10 min, 219 μl 2.5 M KHCO_3_ was added to the supernatant. After centrifuging at 4500 rpm, 4 °C for 2 min, the supernatant was stored at −80 °C. For LC-MS/MS analysis, samples were thawed on ice and centrifuged at 13,000 rpm, 4 °C for 10 min. The supernatant was filtered with a 22 μm filter membrane and 20 μl of the sample was subjected to AB SCIEX 3200 QTRAP (Sciex) for separation using a 4.6 × 50 mm ZORBAX SB-Aq (Agilent). Phase A (Mili-Q H_2_O) and phase B (100% acetonitrile) were used as mobile phases.

### RNA-seq analysis

Total RNA of biofilm harvested from cells incubated statically for 24 h was extracted using TaKaRa MiniBEST Universal RNA Extraction Kit (TaKaRa). Removal of rRNA was performed using MICROBExpress Bacterial mRNA Enrichment Kit (Invitrogen). The rRNA-depleted sample was processed for library preparation with NEBNext Ultra II RNA Library Prep Kit for Illumina (NEB). RNA-seq reads were mapped to ATCC 27853 genome (GenBank: CP011857.1) using TopHat (version 2.1.1) (57). The uniquely mapped reads in this step were used for analysis. Cufflinks (version 2.2.0) (58) was used to identify differentially expressed genes. RNA-seq data are listed in Data. S1.

### Characterizing membrane perturbation using the PI-BactD probe

The membrane feature of *P. aeruginosa* ATCC 27853 and PAO1 was examined using the membrane-sensitive probe PI-BactD as described previously with slight modifications (41). *P. aeruginosa* ATCC 27853 or PAO1 cells were cultured as described for c-di-GMP quantification. ∼0.5 × 10^9^ cells were harvested, washed twice with 1×PBS at ambient temperature, and resuspended in 1 ml 1×PBS (∼8 min for these steps). Prior to adding the PI-BactD probe, the cell suspension was incubated at RT (for cells harvested from RT culture) or 37 °C (for cells harvested from 37 °C culture) for 5 min, respectively. PI-BactD probe was added to the final concentration as 5 μM, and the samples were incubated at RT (for samples harvested from RT culture) or 37 °C (for samples harvested from 37 °C culture) for 1 min. Hundred microliter of the mixture was transferred to each well of a black 96-well plate. The fluorescent spectra of 365 to 650 nm (2 nm interval) was measured with λex = 345 nm using a Thermo Scientific Varioskan Flash spectral scanning multi-technology microplate reader (Thermo Fisher Scientific) at the ambient temperature (25 °C).

### PhosTag PAGE

PhosTag PAGE was conducted following our previous description with slight modification (59). *P. aeruginosa* ATCC 27853 cells containing chromosomal SiaC-3 × *flag* were cultured as described for c-di-GMP quantification. ∼0.5 × 10^9^ cells were pelleted, washed twice with ice-cold 1×PBS, and stored at −80 °C. Frozen pellets were thawed on ice and resuspended in ice-cold 1×sample buffer (13 mM Tris, 0.2% SDS, 5% glycerol, 0.004% bromophenol blue, and pH 6.8) before cell lysis by sonication. Then 25 μl of protein samples was loaded on 10% Tris–Gly polyacrylamide gel containing 25 mM Phos-tag acrylamide (Fujifilm Wako) and 50 mM MnCl_2_. After electrophoresis at 4 °C for 2.5 h, the gel was washed twice with transfer buffer containing 10 mM EDTA and subsequently transferred onto a polyvinylidene difluoride membrane. Membranes were subjected to standard immunoblotting as described in the supplementary information.

### Chromosomal 3×*flag* tagging

To construct chromosomal 3×*flag* tag of *pslD* and *siaC*, ∼500 bp DNA fragment upstream of their stop codon was amplified by PCR and cloned to pAY3400 at the NotI site. The upstream fragment followed by 3×*flag* tag sequence was then amplified from the pAY3400 derivatives. Approximately, 500 bp fragment downstream of *pslD* or *siaC* was amplified from *P. aeruginosa* genomic DNA. The two fragments were cloned to pEX18ApGW at the KpnI and HindIII sites. 3×*flag* tag sequence was integrated to the C terminal of *pslD* or *siaC* in the chromosome of *P. aeruginosa* ATCC 27853 following a previous description (60). To construct chromosomal 3×*flag* tagged *siaA* and the *siaA* variant, the 3×*flag* tag sequence was amplified from pAY3400 and cloned to pJM253 (61) at the SalI site, yielding pAY9656. The promoter region of *sia* and the coding region of *siaA* or the *siaA* variant was amplified and cloned into pAY9656 at the HindIII site. The 3×*flag* tagged *siaA* or the *siaA* variant were integrated at *attB* site of ATCC 27853 Δ*siaA* cells following the previous description (61).

### Western immunoblotting

Overnight culture of *P. aeruginosa* ATCC 27853 cells carrying a chromosomal 3×*flag* tag to the C terminus of PslD, SiaC, or SiaA cultured as described for c-di-GMP quantification. ∼1 × 10^9^ cells were pelleted and subjected to lysis using the B-PER Complete Bacterial Protein Extraction Reagent (Thermo Fisher Scientific). Protein concentration was determined with BCA Protein Quantification Kit (Abcam). Protein samples were separated on a 10% SDS polyacrylamide gel and transferred to a polyvinylidene difluoride membrane. Immunoblotting was carried out using monoclonal anti-FLAG antibody (Sigma) or monoclonal anti-RNA polymerase beta antibody (Abcam) as primary antibodies and HRP conjugate antibodies (BioRad) as secondary antibodies. Blots were treated by ECL Prime Western Blotting Detection Reagent (Cytiva) and the protein bands were visualized utilizing an Alliance Q9 Gel Documentation System (UVTEC). ImageJ software was used to evaluate the protein levels.

### Psl immunoblots

Psl immunoblots were performed as reported previously (62, 63). Static bacteria culture at indicated time points were spun down at 16,000 × *g* for 5 min. Supernatant containing extracellular Psl was collected and treated with 0.5 mg/ml proteinase K at 60 °C for 1 h followed by incubation at 85 °C for 30 min to inactivate proteinase. The samples were then applied to a 0.45 μm nitrocellulose membrane using Bio-Dot 96-Well Microfiltration system (Bio-Rad). After blocking with 10% skim milk, immunoblotting was carried out using anti-Psl antiserum produced in rabbit (1:2000 in 1% skim milk in TBST) (63) and HRP conjugate goat anti-rabbit antibody (1:5000 in 1% skim milk in TBST) (BioRad, 1706515) as secondary antibody. Blots were treated by ECL Prime Western Blotting Detection Reagent (Cytiva) and visualized utilizing an Alliance Q9 Gel Documentation System (UVTEC). ImageJ software was used to evaluate Psl levels.

### Construction of gene deletion mutants

Seamless gene deletions were performed using the two-step allelic exchange method as reported previously (60). Five hundred to eight hundred bp DNA fragments flanking the upstream and downstream of the gene to be deleted were amplified and cloned to the suicide vector pEX18ApGW at the KpnI and HindIII sites. The resulting pEX18ApGW derivatives were transformed to *E. coli* SM10*λpir* and were then mobilized to *P. aeruginosa* by conjugation. Merodiploids were selected on Vogel-Bonner minimal medium agar containing 300 μg/ml carbenicillin, followed by counterselection on NSLB agar supplied with 15% (wt/vol) sucrose. Mutations were confirmed by PCR and Sanger sequencing (BGI Sanger Sequencing, BGI).

### Rhamnose induction

DNA fragment of a gene of interest was amplified and cloned into the vector pJM253 at the HindIII site (61). The *rhaSR*-*PrhaBAD-siaB* cassette or *rhaSR*-*PrhaBAD-fabAB* cassette was integrated at *attB* site of ATCC 27853 or its isogenic mutant cells following the previous description (61).

### Construction of CusS-SiaA chimeric variants

To construct P*sia*-*CusS-T*_*1*-*2*_-*siaA*, the promoter region and the region from R337 to the stop codon of *siaA* were amplified by PCR using the genomic DNA of ATCC 27853 as the template. DNA fragment encoding the N terminus to TM2 region of CusS was amplified from the genomic DNA of *E. coli* MG1655. These three fragments were cloned to the vector pJM253 at the HindIII site, yielding pAY9642. The sequence expressing the SiaA variant was integrated into the *attB* site of ATCC 27853 Δ*siaA* as described above.

### Construction of the SiaC^T68A^ and SiaC^T68D^

To construct ATCC 27853 mutants carrying an allele that encodes SiaC^T68A^ or SiaC^T68D^, DNA fragments encompassing 600 to 700 bp upstream and downstream of T68 amino acid in SiaA were amplified by PCR (Table. S3) and were subsequently cloned to the suicide vector pEX18ApGW at the KpnI and HindIII sites. Introducing *siaC*^*T68A*^ or *siaC*^*T68D*^ to the chromosome of ATCC 27853 was performed by the two-step allelic exchange approach as described above using ATCC 27853 Δ*siaC* as the recipient strain.

### Lipidomics analysis

Thirty to fifty milligrams of biofilm sample grown at RT and 37 °C was collected and washed twice with 0.9% saline. The lipid composition was analyzed using the lipidomics service at Proteomics and Metabolomics Core, HKU. For LC-MS/MS sample preparation, chloroform:methanol (2:1, v/v) was added to the cell pellet and followed by sonification. After centrifuging at 3000 g for 5 min, the supernatant was aliquot and dried under nitrogen. Then the sample was reconstituted with IPA:methanol:chloroform (1:1:0.2, v/v) and subjected to the LC-MS/MS system (Vanquish UPLC, Thermo Fisher Scientific) using a Accucore C30 (2.1 × 150 mm, 2.6 μm) (Thermo Fisher Scientific). Phase A (10 mM ammonium formate with 0.1% formic acid in acetonitrile and water, v/v 6:4) and phase B (10 mM ammonium formate with 0.1% formic acid in acetonitrile and IPA 1:9) were used as mobile phases. The mass spectrometry analysis was processed using an Orbitrap Exploris 120 mass spectrometer (Thermo Fisher Scientific) equipped with a HESI II probe. Data analysis was performed using Lipidsearch (Thermofisher Scientific/Mitsui Knowledge Industries). Raw peak areas for all identified lipids were obtained, and the relative area of lipid groups was calculated by dividing the total area at RT by that of 37 °C. Lipidomics analysis data are listed in Data. S2.

### Quantification and statistical analysis

Data represent the mean ± SD of at least three biological replicates (independent, fresh bacterial colonies). Statistical analysis was performed by Student’s *t* test (2-tailed) in Microsoft Excel. *p* values less than 0.05 were considered significant.

## Data availability

The data generated and analyzed during the current study are available from the corresponding author upon reasonable request.

## Supporting information

This article contains supporting information (23, 26, 55, 61, 64, 65).

## Conflict of interest

The authors declare that they have no conflicts of interests with the contents of this article.
