# Supplementary material for: Temperature downshifts induce biofilm formation in Pseudomonas aeruginosa through the SiaABCD signal and functional module
Source: J Biol Chem. 2025 Dec 22;302(2):111086. doi: 10.1016/j.jbc.2025.111086 (PMC12834913; doi:10.1016/j.jbc.2025.111086)
Supplement: Supplementary Tables and Figures [file mmc3.pdf]

Supplementary Information

Temperature downshifts induce biofilm formation in *Pseudomonas aeruginosa* through the SiaABCD signal and functional module

Yanran Li[1], Zhe Chen[1], Tingying Xia[1], Yiqing Ding[2], Yingpeng Xie[2], Lu Miao[3], Zhaochao Xu[3], Xin Deng[2], Luyan Z. Ma[4] and Aixin Yan[1*]

[1] School of Biological Sciences, The University of Hong Kong, Pokfulam Road, Hong Kong SAR, China

[2] Department of Biomedical Sciences, City University of Hong Kong, Kowloon Tong, Hong Kong SAR, China

[3] CAS Key Laboratory of Separation Science for Analytical Chemistry, Dalian Institute of Chemical Physics, Chinese Academy of Sciences, Dalian 116023, China

[4] State Key Laboratory of Microbial Resources, Institute of Microbiology, Chinese Academy of Sciences, Beijing, China

* Correspondence: ayan8@hku.hk (A.Y.)

Table of contents

Fig. S1 to S12

Table S1 to S6

Data S1 to S2

Supplementary Figures

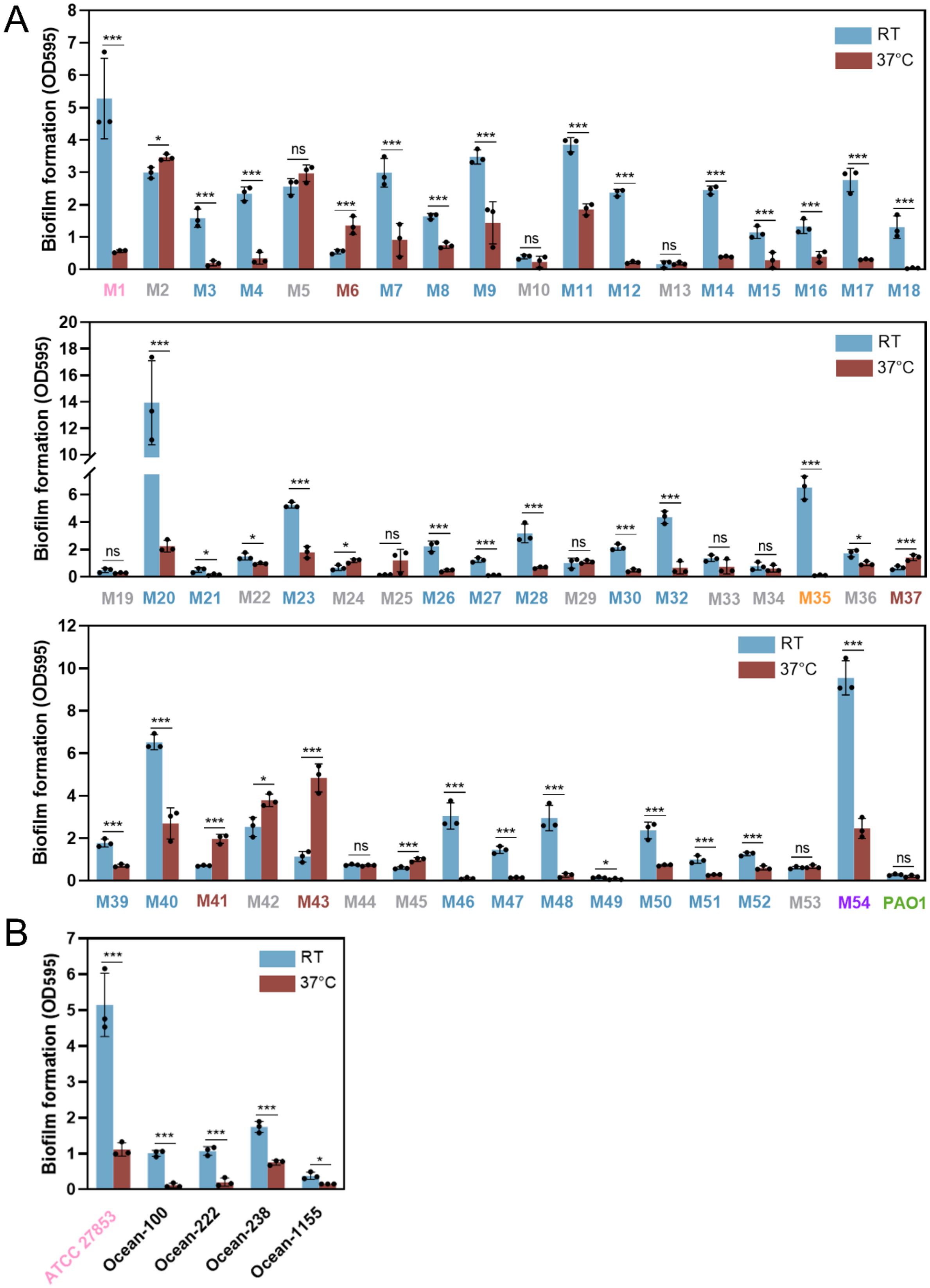

Fig S1. Biofilm formation patterns of 52 clinical and 4 environmental *P. aeruginosa* isolates. **A**, Bar graphs of biofilm formation of 52 clinical isolates and reference strain PAO1 at RT and 37°C for 48 h. Data are represented as mean ± SD, n = 3 independent experiments. **B**, Biofilm biomass of environmental isolates Ocean-100, Ocean-222, Ocean-238, and Ocean-1155 at RT and 37°C for 48 h. The strain names are colored according to the corresponding colors in Fig. 1. Blue: strains formed two-fold or more biofilm at RT relative to 37°C. Red: strains formed two-fold or more biofilm at 37°C relative to RT. Black: environmental isolates. Pink: M1 (ATCC 27853). Orange: M35 (PA150663). Purple: M54 (PA152541). Green: PAO1. Gray: strains formed similar levels of biofilm at RT and 37°C. Data are represented as mean ± SD, n = 3 independent experiments. *, $p < 0.05$. ***, $p < 0.01$. ns, not significant (based on Student's *t* test).

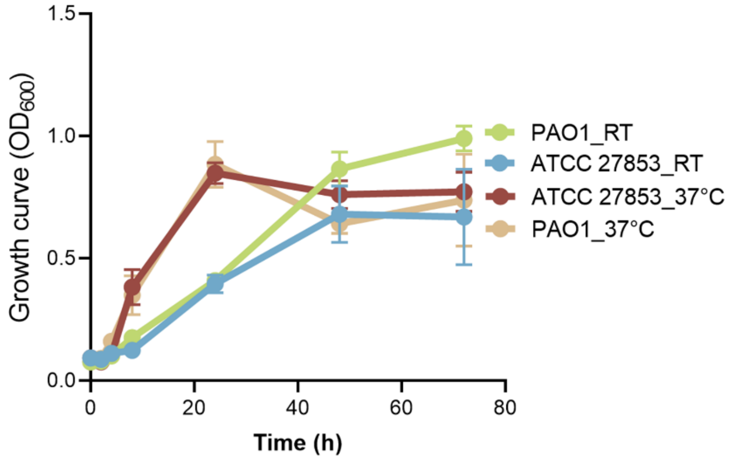

Fig S2. Growth curve of ATCC 27853 and PAO1 standing culture at RT and 37°C. Data are represented as mean ± SD, n = 3 independent experiments. No significant differences were found between PAO1 and ATCC 27853 at RT or 37°C (based on Student's *t* test).

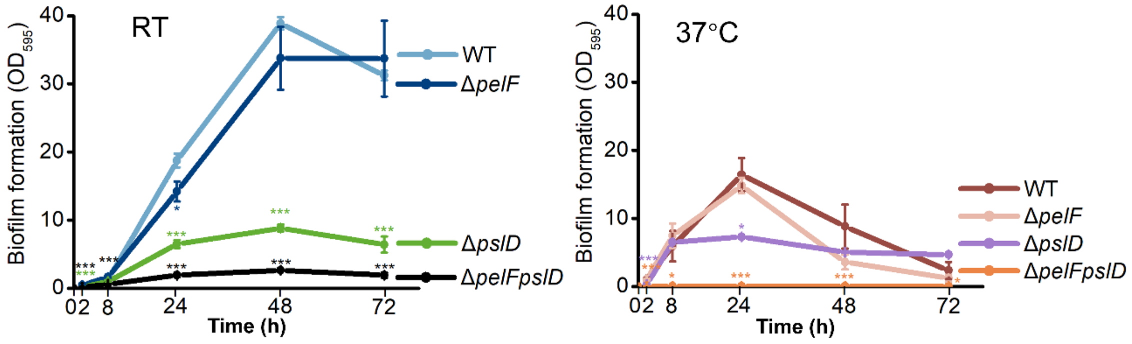

Fig S3. Dynamic biofilm formation of ATCC 27853 and its isogenic Δ*pslD*, Δ*pelF*, Δ*pslD*Δ*pelF* strain at RT and 37°C. Data are represented as mean ± SD, n = 3 independent experiments. *, $p < 0.05$. ***, $p < 0.01$ (based on Student's *t* test).

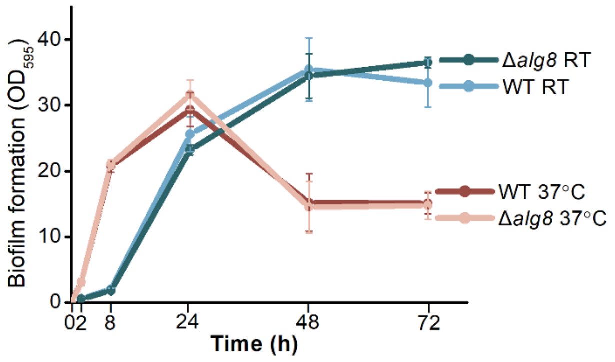

Fig S4. Dynamic biofilm formation of ATCC 27853 and its isogenic Δ*alg8* strain at RT and 37°C. Data are represented as mean ± SD, n = 3 independent experiments. No significant differences were found between ATCC 27853 and its isogenic Δ*alg8* strain RT or 37°C (based on Student's *t* test).

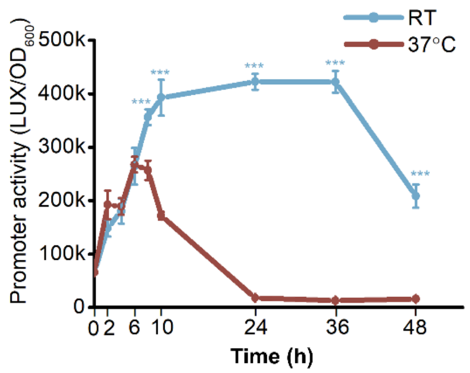

Fig S5. Activity of chromosomal P*psl-lux* transcriptional fusion reporters in ATCC 27853 at RT and 37°C. Data are represented as mean ± SD, n = 3 independent experiments. *, $p < 0.05$. ***, $p < 0.01$ (based on Student's *t* test).

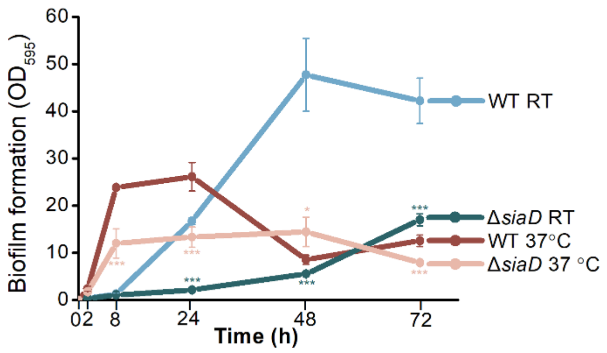

Fig S6. Dynamic biofilm formation of ATCC 27853 and its isogenic Δ*siaD* strain at RT and 37°C. Data are represented as mean ± SD, n = 3 independent experiments. *, $p < 0.05$. ***, $p < 0.01$ (based on Student's *t* test).

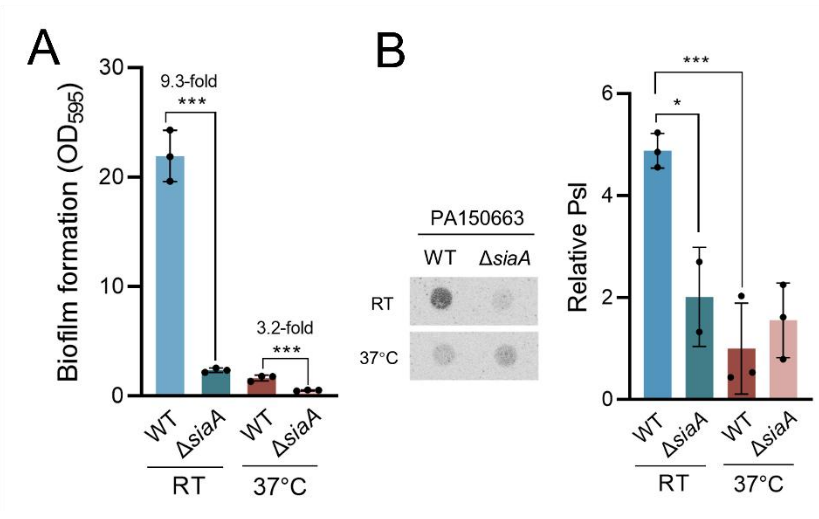

Fig S7. A, Biofilm biomass of PA150663 and its isogenic Δ*siaA* mutants at RT and 37°C at 48 h. Data are represented as mean ± SD, n = 3 independent experiments. **B**, Relative Psl production in the biofilm matrix of PA150663 and its isogenic Δ*siaA* mutant at RT and 37°C as detected by Psl immunobloting. Data are represented as mean ± SD, n = 3 independent experiments. ***, $p < 0.01$ (based on Student's *t* test).

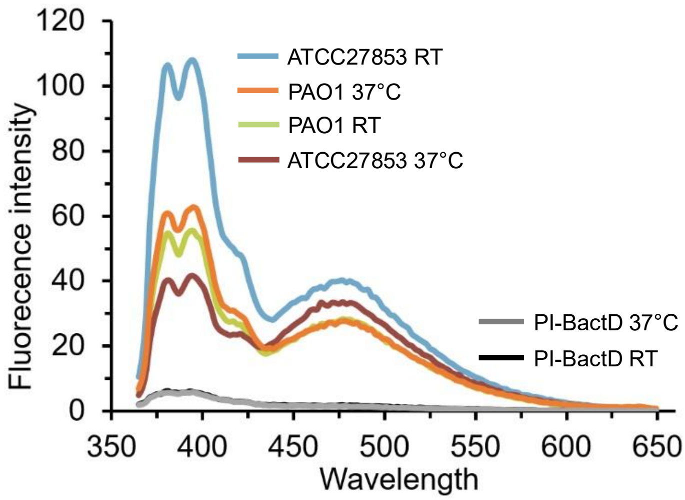

Fig S8. Fluorescence spectra of PI-BactD treated ATCC 27853 or PAO1 cells grown at RT or 37°C. Data are presented as the mean of six replicates.

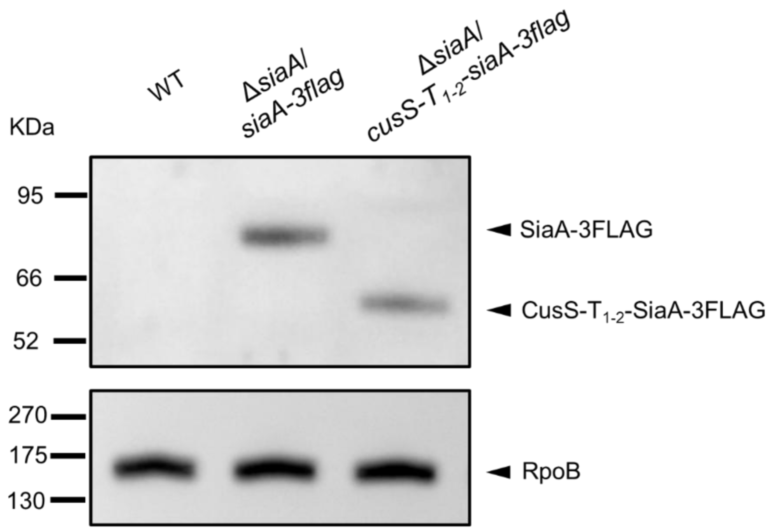

Fig S9. Western blot of SiaA-3FLAG and CusS-$T_{1\text{-}2}$-SiaA-3FLAG in ATCC 27853 isogenic Δ*siaA* mutant cultured at RT.

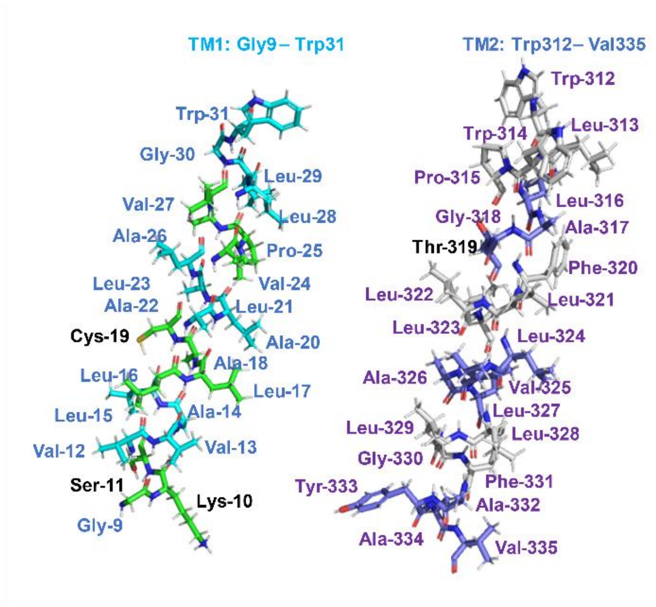

Fig S10. Predicted transmembrane region of SiaA: TM1 (Gly9–Trp31) and TM2 (Trp312–Val335). Hydrophilic residues are highlighted in black; hydrophobic residues are highlighted in blue (TM1) and purple (TM2).

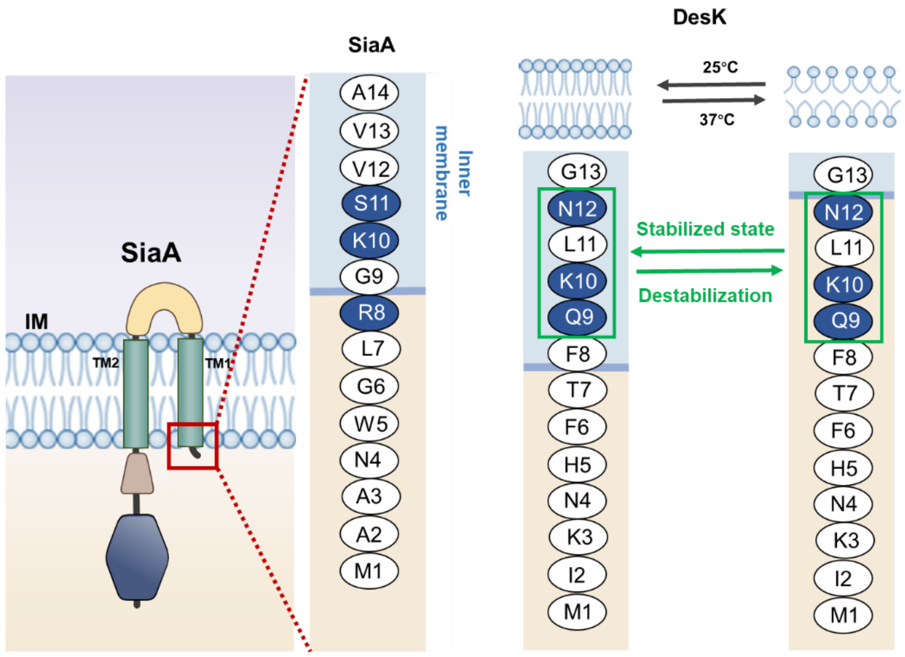

Fig S11. Schematic representation of the N-terminal region of *P. aeruginosa* ATCC 27853 SiaA (left panel) and *B. subtilis* JH642 DesK (right panel). Blue: hydrophilic amino acids. Green box: sunken buoy (SB) motif. In the “sunken buoy” model, SB motif is in a hydrated/stabilized state at 37°C and in dehydration/destabilization at lowered temperature.

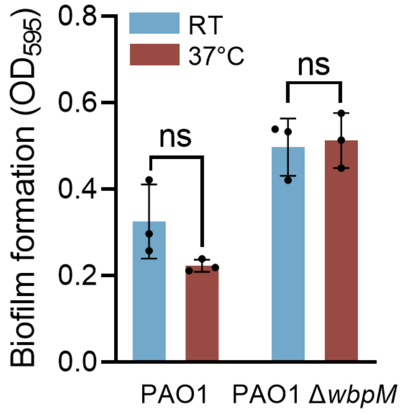

Fig S12. Biofilm biomass of PAO1 and its isogenic Δ*wbpM* mutant at RT and 37°C. Data are represented as mean ± SD, n = 3 independent experiments. ns, not significant (based on Student’s *t* test).

Supplementary Tables

Table. S1 Bacterial strains used in this study

Strain	Description	Reference
E. coli		
DH5α	F– φ80*lacZΔM15* Δ(*lacZYA-argF*)*U169 recA1 endA1 hsdR17*(rK⁻, mK⁺) *phoA supE44 λ–thi-1 gyrA96 relA1*	Lab collection
SM10*λpir*	*thi thr leu tonA lacY supE recA::RP4-2-Tc::Mu Km λpir*	Lab collection
***P. aeruginosa* ATCC 27853**		
ATCC 27853	Wild-type strain	Lab collection
PA27853*ΔlasR*	PA27853 Δ*lasR*	Lab collection
PA27853*ΔpqsA*	PA27853 Δ*pqsA*	Lab collection
PA27853*ΔrhlR*::Gm	PA27853 Δ*rhlR*::*Gm*	Lab collection
AY6603	ATCC 27853 Δ*pslD*	This study
AY6647	ATCC 27853 Δ*fleQ*	This study
AY6648	ATCC 27853 Δ*rocR*	This study
AY6649	ATCC 27853 Δ*amrZ*	This study
AY6652	ATCC 27853 Δ*rpoS*	This study
AY6685	ATCC 27853 Δ*PA5295*	This study
AY7627	ATCC 27853 Δ*pelF*	This study
AY7629	ATCC 27853 Δ*pslD*Δ*pelF*	This study
AY7695	ATCC 27853 Δ*PA2572*	This study
AY7696	ATCC 27853 Δ*PA4108*	This study
AY7697	ATCC 27853 Δ*PA4781*	This study

AY7700	ATCC 27853 Δ*rbdA*	This study
AY7701	ATCC 27853 Δ*PA1433*	This study
AY7702	ATCC 27853 Δ*dipA*	This study
AY7714	ATCC 27853 Δ*roeA*	This study
AY7715	ATCC 27853 Δ*morA*	This study
AY7718	ATCC 27853 Δ*gcbA*	This study
AY7736	ATCC 27853 Δ*PA1851*	This study
AY7738	ATCC 27853 Δ*PA2567*	This study
AY7739	ATCC 27853 Δ*sadC*	This study
AY7740	ATCC 27853 Δ*fimX*	This study
AY7663	ATCC 27853 Δ*wspF*	This study
AY7765	ATCC 27853 Δ*siaD*	This study
AY7766	ATCC 27853 Δ*wspR*	This study
AY7779	ATCC 27853 Δ*toxR*	This study
AY7788	ATCC 27853 *pslD*-3*flag*	This study
AY9559	ATCC27853 *attB"P*::P*psl-lux*Δ*FRT*	This study
AY9564	ATCC27853 Δ*siaD attB"P*::P*psl-lux*Δ*FRT*	This study
AY9565	ATCC27853 Δ*fleQ attB"P*::P*psl-lux*Δ*FRT*	This study
AY9571	ATCC 27853 Δ*siaB*	This study
AY9572	ATCC 27853 Δ*siaC*	This study
AY9593	ATCC 27853 Δ*siaA*	This study
AY9604	ATCC 27853 *attB"P*::pJM253Δ*FRT*	This study
AY9605	ATCC 27853 *attB"P*::(pJM253-*siaB*)Δ*FRT*	This study
AY9606	ATCC 27853 Δ*siaB attB"P*::pJM253Δ*FRT*	This study
AY9607	ATCC 27853 Δ*siaB attB"P*::(pJM253-*siaB*)Δ*FRT*	This study
AY9622	ATCC 27853 *siaC*-3*flag*	This study
AY9643	ATCC 27853 Δ*siaA attB*"*P*::(pJM253::P*siaA-siaA*)Δ*FRT*	This study
AY9653	ATCC 27853 Δ*siaA attB*"*P*::(pJM253::P*siaA-cus*T_{1-2}-*siaA*)Δ*FRT*	This study

AY9682	ATCC 27853 Δ*siaA attB*"*P*::(pAY9656::P*siaA-siaA*)Δ*FRT*	This study
AY9683	ATCC 27853 Δ*siaA attB*"*P*::(pAY9656::P*siaA-cusS-*$T_{1\text{-}2}$*-siaA*)Δ*FRT*	This study
AY9695	PA150663 (M35) Δ*siaA*	This study
AY9697	ATCC 27853 Δ*siaD pslD*-3*flag*	This study
AY9710	ATCC 27853 *attB*"*P*::(pJM253_P*rha-fabAB*)Δ*FRT*	This study
AY9720	ATCC 27853 $siaC^{T68A}$	This study
AY9721	ATCC 27853 $siaC^{T68D}$	This study
AY9766	ATCC 27853 Δ*alg8*	This study
AY9782	ATCC 27853 Δ*Ppsl*	This study
AY11012	ATCC 27853 Δ*siaD attB"P*::(pJM253-*siaD*)	This study
AY11015	ATCC 27853 *attB"P*::pJM253	This study

P. aeruginosa **PAO1**

PAO1	Wild-type strain	Lab collection
PAO1	PAO1 Δ*wbpM*	This study

P. aeruginosa **isolates**

PA130788 (M2)	*P. aeruginosa* isolate from blood, Queen Mary Hospital (HK)	Lab collection
PA132526 (M3)	*P. aeruginosa* isolate from blood, Queen Mary Hospital (HK)	Lab collection
PA132533 (M4)	*P. aeruginosa* isolate from blood, Queen Mary Hospital (HK)	Lab collection
PA139140 (M5)	*P. aeruginosa* isolate from blood, Queen Mary Hospital (HK)	Lab collection
PA147513 (M6)	*P. aeruginosa* isolate from blood, Queen Mary Hospital (HK)	Lab collection
PA149218 (M7)	*P. aeruginosa* isolate from blood, Queen Mary Hospital (HK)	Lab collection
PA149488 (M8)	*P. aeruginosa* isolate from blood, Queen Mary Hospital (HK)	Lab collection
PA150567 (M9)	*P. aeruginosa* isolate from blood, Queen Mary Hospital (HK)	Lab collection
PA150577 (M10)	*P. aeruginosa* isolate from blood, Queen Mary Hospital (HK)	Lab collection

PA150873 (M11)	*P. aeruginosa* isolate from blood, Queen Mary Hospital (HK)	Lab collection
PA151514 (M12)	*P. aeruginosa* isolate from blood, Queen Mary Hospital (HK)	Lab collection
PA151671 (M13)	*P. aeruginosa* isolate from blood, Queen Mary Hospital (HK)	Lab collection
PA151908 (M14)	*P. aeruginosa* isolate from blood, Queen Mary Hospital (HK)	Lab collection
PA151970 (M15)	*P. aeruginosa* isolate from blood, Queen Mary Hospital (HK)	Lab collection
PA151971 (M16)	*P. aeruginosa* isolate from blood, Queen Mary Hospital (HK)	Lab collection
PA152165 (M17)	*P. aeruginosa* isolate from blood, Queen Mary Hospital (HK)	Lab collection
PA152241 (M18)	*P. aeruginosa* isolate from blood, Queen Mary Hospital (HK)	Lab collection
PA152603 (M19)	*P. aeruginosa* isolate from blood, Queen Mary Hospital (HK)	Lab collection
PA152821 (M20)	*P. aeruginosa* isolate from blood, Queen Mary Hospital (HK)	Lab collection
PA153195 (M21)	*P. aeruginosa* isolate from blood, Queen Mary Hospital (HK)	Lab collection
PA153306 (M22)	*P. aeruginosa* isolate from blood, Queen Mary Hospital (HK)	Lab collection
PA153541 (M23)	*P. aeruginosa* isolate from blood, Queen Mary Hospital (HK)	Lab collection
PA153837 (M24)	*P. aeruginosa* isolate from blood, Queen Mary Hospital (HK)	Lab collection
PA153983 (M25)	*P. aeruginosa* isolate from blood, Queen Mary Hospital (HK)	Lab collection
PA154054 (M26)	*P. aeruginosa* isolate from blood, Queen Mary Hospital (HK)	Lab collection
PA154197 (M27)	*P. aeruginosa* isolate from blood, Queen Mary Hospital (HK)	Lab collection
PA129904-4 (M28)	*P. aeruginosa* isolate from blood, Queen Mary Hospital (HK)	Lab collection
PA139357-2 (M29)	*P. aeruginosa* isolate from blood, Queen Mary Hospital (HK)	Lab collection
PA149623-2 (M30)	*P. aeruginosa* isolate from blood, Queen Mary Hospital (HK)	Lab collection
PA150209-1 (M31)	*P. aeruginosa* isolate from blood, Queen Mary Hospital (HK)	Lab collection
PA150210-1 (M32)	*P. aeruginosa* isolate from blood, Queen Mary Hospital (HK)	Lab collection
PA150317-1 (M33)	*P. aeruginosa* isolate from blood, Queen Mary Hospital (HK)	Lab collection
PA150571-1 (M34)	*P. aeruginosa* isolate from blood, Queen Mary Hospital (HK)	Lab collection
PA150663 (M35)	*P. aeruginosa* isolate from blood, Queen Mary Hospital (HK)	Lab collection
PA151345-3 (M36)	*P. aeruginosa* isolate from blood, Queen Mary Hospital (HK)	Lab collection
PA152211-1 (M37)	*P. aeruginosa* isolate from blood, Queen Mary Hospital (HK)	Lab collection
PA152211-2 (M38)	*P. aeruginosa* isolate from blood, Queen Mary Hospital (HK)	Lab collection

PA152361-1 (M39)	*P. aeruginosa* isolate from blood, Queen Mary Hospital (HK)	Lab collection
PA152936-1 (M40)	*P. aeruginosa* isolate from blood, Queen Mary Hospital (HK)	Lab collection
PA153212-1 (M41)	*P. aeruginosa* isolate from blood, Queen Mary Hospital (HK)	Lab collection
PA153478-2 (M42)	*P. aeruginosa* isolate from blood, Queen Mary Hospital (HK)	Lab collection
PA153533-1 (M43)	*P. aeruginosa* isolate from blood, Queen Mary Hospital (HK)	Lab collection
PA153536-2 (M44)	*P. aeruginosa* isolate from blood, Queen Mary Hospital (HK)	Lab collection
PA153543-2 (M45)	*P. aeruginosa* isolate from blood, Queen Mary Hospital (HK)	Lab collection
PA153545-3 (M46)	*P. aeruginosa* isolate from blood, Queen Mary Hospital (HK)	Lab collection
PA153673-2 (M47)	*P. aeruginosa* isolate from blood, Queen Mary Hospital (HK)	Lab collection
PA154019-1 (M48)	*P. aeruginosa* isolate from blood, Queen Mary Hospital (HK)	Lab collection
PA154283-1 (M49)	*P. aeruginosa* isolate from blood, Queen Mary Hospital (HK)	Lab collection
PA154367 (M50)	*P. aeruginosa* isolate from blood, Queen Mary Hospital (HK)	Lab collection
PA151831-4 (M51)	*P. aeruginosa* isolate from blood, Queen Mary Hospital (HK)	Lab collection
PA152068-3 (M52)	*P. aeruginosa* isolate from blood, Queen Mary Hospital (HK)	Lab collection
PA152515 (M53)	*P. aeruginosa* isolate from blood, Queen Mary Hospital (HK)	Lab collection
PA152541 (M54)	*P. aeruginosa* isolate from blood, Queen Mary Hospital (HK)	Lab collection
Ocean-100	Wild-type strain	Kumagai et al.(1)
Ocean-222	Wild-type strain	Kumagai et al.(1)
Ocean-238	Wild-type strain	Kumagai et al.(1)
Ocean-1155	Wild-type strain	Kumagai et al.(1)

Table S2. Plasmids used in this study

Plasmids	Description	Reference
pEX18ApGW	Gateway cloning vector. *att*R1 and *att*R2 sites, *oriT*+, *ccdB*+, *sacB*+. Amp^R	Choi et al. (2)
pFLP2	Express Flp recombinase. Amp^R	Hoang et al. (3)
pJM253	Integration vector for *P. aeruginosa. rhaSR-PrhaBAD* inducible promoter. Tc^R	Meisner et al. (4)
pmini-CTX-*lux*	Integration vector for *P. aeruginosa*. Used for engineering of *lux* reporter and expression in strains. *attP* site, *FRT* sites, *luxCDABE*, *ori*, *oriT*. Tc^R	Becher et al. (5)
pAY3400	pBSK-ICmI-3FLAG. Cm^R	Lab collection
pAY5792	*pslD* deletion cassette cloned to pEX18ApGW using KpnI and HindIII restriction sites. Gm^R, Amp^R	This study
pAY6637	*amrZ* deletion cassette cloned to pEX18ApGW using KpnI and HindIII restriction sites. Gm^R, Amp^R	This study
pAY6638	*fleQ* deletion cassette cloned to pEX18ApGW using KpnI and HindIII restriction sites. Gm^R, Amp^R	This study
pAY6639	*rpoS* deletion cassette cloned to pEX18ApGW using KpnI and HindIII restriction sites. Gm^R, Amp^R	This study
pAY6640	*rocR* deletion cassette cloned to pEX18ApGW using KpnI and HindIII restriction sites. Gm^R, Amp^R	This study
pAY6662-2	*PA5295* deletion cassette cloned to pEX18ApGW using KpnI and HindIII restriction sites. Gm^R, Amp^R	This study
pAY7623	*pelF* deletion cassette cloned to pEX18ApGW using KpnI and HindIII restriction sites. Gm^R, Amp^R	This study
pAY7656	*wspF* deletion cassette cloned to pEX18ApGW using KpnI and HindIII restriction sites. Gm^R, Amp^R	
pAY7665	*pslD* 3*flag* tagging cassette cloned to pEX18ApGW using KpnI and HindIII restriction sites. Gm^R, Amp^R	This study
pAY7678	*PA2572* deletion cassette cloned to pEX18ApGW using KpnI and HindIII restriction sites. Gm^R, Amp^R	This study

pAY7679	*PA4108* deletion cassette cloned to pEX18ApGW using KpnI and HindIII restriction sites. Gm^R, Amp^R	This study
pAY7680	*PA*4781deletion cassette cloned to pEX18ApGW using KpnI and HindIII restriction sites. Gm^R, Amp^R	This study
pAY7685	P*psl* cloned to pmini-CTX-*lux* using HindIII restriction site. Tc^R	This study
pAY7689	*rbdA* deletion cassette cloned to pEX18ApGW using KpnI and HindIII restriction sites. Gm^R, Amp^R	This study
pAY7690	*PA1433* deletion cassette cloned to pEX18ApGW using KpnI and HindIII restriction sites. Gm^R, Amp^R	This study
pAY7691	*dipA* deletion cassette cloned to pEX18ApGW using KpnI and HindIII restriction sites. Gm^R, Amp^R	This study
pAY7703	*gcbA* deletion cassette cloned to pEX18ApGW using KpnI and HindIII restriction sites. Gm^R, Amp^R	This study
pAY7703	*roeA* deletion cassette cloned to pEX18ApGW using KpnI and HindIII restriction sites. Gm^R, Amp^R	This study
pAY7704	*morA* deletion cassette cloned to pEX18ApGW using KpnI and HindIII restriction sites. Gm^R, Amp^R	This study
pAY7724	*PA1851* deletion cassette cloned to pEX18ApGW using KpnI and HindIII restriction sites. Gm^R, Amp^R	This study
pAY7725	*fimX* deletion cassette cloned to pEX18ApGW using KpnI and HindIII restriction sites. Gm^R, Amp^R	This study
pAY7727	*PA2567* deletion cassette cloned to pEX18ApGW using KpnI and HindIII restriction sites. Gm^R, Amp^R	This study
pAY7728	*PA2072* deletion cassette cloned to pEX18ApGW using KpnI and HindIII restriction sites. Gm^R, Amp^R	This study
pAY7730	*sadC* deletion cassette cloned to pEX18ApGW using KpnI and HindIII restriction sites. Gm^R, Amp^R	This study
pAY7753	*wspR* deletion cassette cloned to pEX18ApGW using KpnI and HindIII restriction sites. Gm^R, Amp^R	This study
pAY7760	*siaD* deletion cassette cloned to pEX18ApGW using KpnI and HindIII restriction sites. Gm^R, Amp^R	This study

pAY7771	*toxR* deletion cassette cloned to pEX18ApGW using KpnI and HindIII restriction sites. Gm^R, Amp^R	This study
pAY9532	P*sia* cloned to pmini-CTX-*lux* using HindIII restriction site. Tc^R	This study
pAY9546	*siaB* deletion cassette cloned to pEX18ApGW using KpnI and HindIII restriction sites. Gm^R, Amp^R	This study
pAY9547	*siaC* deletion cassette cloned to pEX18ApGW using KpnI and HindIII restriction sites. Gm^R, Amp^R	This study
pAY9579	*siaA* deletion cassette cloned to pEX18ApGW using KpnI and HindIII restriction sites. Gm^R, Amp^R	This study
pAY9587	*siaB* cloned to pJM253 using SpeI restriction site. Tc^R	This study
pAY9616	*siaC* 3*flag* tagging cassette cloned to pEX18ApGW using KpnI and HindIII restriction sites. Gm^R, Amp^R	This study
pAY9642	P*sia-cusS-*$T_{1\text{-}2}$*-siaA* cloned to pJM253 using HindIII restriction site. Tc^R	This study
pAY9656	3*flag* inserted into pJM253 using SalI restriction site. Tc^R	This study
pAY9669	P*sia-siaA* cloned to pAY9656 using HindIII restriction site. Tc^R	This study
pAY9670	P*sia-cusS-*$T_{1\text{-}2}$*-siaA* cloned to pAY9656 using HindIII restriction site. Tc^R	This study
pAY9691	PA150663 (M35) *siaA* deletion cassette cloned to pEX18ApGW using KpnI and HindIII restriction sites. Gm^R, Amp^R	This study
pAY9705	*fabAB* cloned to pJM253 using HindIII restriction site. Tc^R	This study
pAY9712	$siaC^{T68A}$ construction cassette cloned to pEX18ApGW using KpnI and HindIII restriction sites. Gm^R, Amp^R	This study
pAY9713	$siaC^{T68D}$ construction cassette cloned to pEX18ApGW using KpnI and HindIII restriction sites. Gm^R, Amp^R	This study
pAY9762	*alg8* deletion cassette cloned to pEX18ApGW using KpnI and HindIII restriction sites. Gm^R, Amp^R	This study
pAY9764	*PAO1 wbpM* deletion cassette cloned to pEX18ApGW using KpnI and HindIII restriction sites. Gm^R, Amp^R	This study
pAY9778	P*psl* deletion cassette cloned to pEX18ApGW using KpnI and HindIII restriction sites. Gm^R, Amp^R	This study
pAY9796	P*sia-siaA* cloned to pJM253 using HindIII restriction site. Tc^R	This study

pAY11006	*siaD* cloned to pJM253 using SpeI restriction site. Tc^R	This study

Table S3. Primers used in this study

Primers	Sequence (5' to 3')	Description
*pslD*_UF	CGACGGCCAGTGCCAATGGGTGACCTCGAATTT	Amplify the upstream donor for constructing Δ*pslD*
*pslD*_UR	TACAGGAAGTGCTCCCTCGGAGCGACATCGCCATGA	
*pslD*_DF	TCATGGCGATGTCGCTCCGAGGGAGCACTTCCTGTA	Amplify the downstream donor for constructing Δ*pslD*
*pslD*_DR	ACGAATTCGAGCTCGCTTCAGTTCCGACTCGTT	
*amrZ*_UF	CGACGGCCAGTGCCAAGGAGCGTGGATTTGCCG	Amplify the upstream donor for constructing Δ*amrZ*
*amrZ*_UR	ACGCGTGGGCTTCGGCGCACATTGAACCTGTAGAGT	
*amrZ*_DF	ACTCTACAGGTTCAATGTGCGCCGAAGCCCACGCGT	Amplify the downstream donor for constructing Δ*amrZ*
*amrZ*_DR	ACGAATTCGAGCTCGTGCAACAGATACAGGTAG	
*fleQ*_UF	CGACGGCCAGTGCCAGAGGCATCCCCAGAGGGA	Amplify the upstream donor for constructing Δ*fleQ*
*fleQ*_UR	GCGTTGCGAAACGACCTGTTTGATCAGCTGCCTTGC	
*fleQ*_DF	GCAAGGCAGCTGATCAAACAGGTCGTTTCGCAACGC	Amplify the downstream donor for constructing Δ*fleQ*
*fleQ*_DR	ACGAATTCGAGCTCGAGGTCGTTGAGCAGGATC	
*rpoS*_UF	CGACGGCCAGTGCCAAGAAGGATGCCCTGC	Amplify the upstream donor for constructing Δ*rpoS*
*rpoS*_UR	CATGCAAGGGATAACGACCGGAAAACCTTAGACCCA	
*rpoS*_DF	TGGGTCTAAGGTTTTCCGGTCGTTATCCCTTGCATG	Amplify the downstream donor for constructing Δ*rpoS*
*rpoS*_DR	ACGAATTCGAGCTCGACTCCCGTCGTCGCGGGG	

*rocR*_UF	CGACGGCCAGTGCCAGGCTGAGCACCAGGGAGG	Amplify the upstream donor for constructing Δ*rocR*
*rocR*_UR	AACCCTGTGTGGACCGTGCTCCGGATCCTGACGCCG	
*rocR*_DF	CGGCGTCAGGATCCGGAGCACGGTCCACACAGGGTT	Amplify the downstream donor for constructing Δ*rocR*
*rocR*_DR	ACGAATTCGAGCTCGAGGGACTTGATCCGCGAG	
*PA5295*_UF	CGACGGCCAGTGCCATCGTCGACGCCGTCGTCC	Amplify the upstream donor for constructing Δ*PA5295*
*PA5295*_UR	CCAGCGAGGCGTCGGCGCCGCTGACAAGCAACCTCC	
*PA5295*_DF	GGAGGTTGCTTGTCAGCGGCGCCGACGCCTCGCTGG	Amplify the downstream donor for constructing Δ*PA5295*
*PA5295*_DR	ACGAATTCGAGCTCGCCCTGTGCATGGGCATCA	
*pelF*_UF	CGACGGCCAGTGCCACCGATCAGCAATGCCAGC	Amplify the upstream donor for constructing Δ*pelF*
*pelF*_UR	CCTGGCGAGATACTGGACCATGGCCGGCATCGGCTT	
*pelF*_DF	AAGCCGATGCCGGCCATGGTCCAGTATCTCGCCAGG	Amplify the downstream donor for constructing Δ*pelF*
*pelF*_DR	ACGAATTCGAGCTCGGGACGTCCTGCGCCATGC	
*wpsF*_UF	CGACGGCCAGTGCCACGCCACCAGCTCGATGGC	Amplify the upstream donor for constructing Δ*wspF*
*wpsF*_UR	GATCGGAGAAGCACAGGGCCGGGCGCGAGTCCGGCC	
*wpsF*_DF	GGCCGGACTCGCGCCCGGCCCTGTGCTTCTCCGATC	Amplify the downstream donor for constructing Δ*wspF*
*wpsF*_DR	ACGAATTCGAGCTCGCTGGACGACGGTTCGCCG	
*PA2572*_UF	CGACGGCCAGTGCCATCGCAGATTTCCGACCAC	Amplify the upstream donor for constructing Δ*PA2572*
*PA2572*_UR	CCCGGTGGACAGACACTCGCGGCTCCCGTCAGATCT	
*PA2572*_DF	AGATCTGACGGGAGCCGCGAGTGTCTGTCCACCGGG	Amplify the downstream donor for constructing Δ*PA2572*
*PA2572*_DR	ACGAATTCGAGCTCGGAGGCAAGCTTCTCCGTC	
*PA4108*_UF	CGACGGCCAGTGCCATGGTTGCTGGAGATGGCT	

*PA4108*_UR	CTCGAGGTCGAGCCCATCACGCCTTCCGCCGGCAAG	Amplify the upstream donor for constructing Δ*PA4108*
*PA4108*_DF	CTTGCCGGCGGAAGGCGTGATGGGCTCGACCTCGAG	Amplify the downstream donor for constructing Δ*PA4108*
*PA4108*_DR	ACGAATTCGAGCTCGGGGTTCCGTCACGACTGC	
*PA4781*_UF	CGACGGCCAGTGCCATCCGCCACCAGTTCCATC	Amplify the upstream donor for constructing Δ*PA4781*
*PA4781*_UR	GCGAAGGCCGGAGGGGCGTACGCCGATCGGTGCGCA	
*PA4781*_DF	TGCGCACCGATCGGCGTACGCCCCTCCGGCCTTCGC	Amplify the downstream donor for constructing Δ*PA4781*
*PA4781*_DR	ACGAATTCGAGCTCGGTGTTGCTCGGCACCCTG	
*rbdA*_UF	CGACGGCCAGTGCCAGGGCTTTGCAGATCGCCC	Amplify the upstream donor for constructing Δ*rbdA*
*rbdA*_UR	TCTTCGCCCCGCGGTCACTCCATCTACCATTCAAAC	
*rbdA*_DF	GTTTGAATGGTAGATGGAGTGACCGCGGGGCGAAGA	Amplify the downstream donor for constructing Δ*rbdA*
*rbdA*_DR	ACGAATTCGAGCTCGATGATCTCCAGCGCCTCC	
*PA1433*_UF	CGACGGCCAGTGCCAATTGGTCGGCGCGAAGAG	Amplify the upstream donor for constructing Δ*PA1433*
*PA1433*_UR	GCACCCAAGGAGGATTGATAGCGAGTCCGGAGCGGG	
*PA1433*_DF	CCCGCTCCGGACTCGCTATCAATCCTCCTTGGGTGC	Amplify the downstream donor for constructing Δ*PA1433*
*PA1433*_DR	ACGAATTCGAGCTCGTCGATGTCCCCAACCCCC	
*dipA*_UF	CGACGGCCAGTGCCAGTTCTTCGCCAGGTACTG	Amplify the upstream donor for constructing Δ*dipA*
*dipA*_UR	ACCTGGAATCAGCCTCGCTCGCCCAGGCCTCCCCCG	
*dipA*_DF	CGGGGGAGGCCTGGGCGAGCGAGGCTGATTCCAGGT	Amplify the downstream donor for constructing Δ*dipA*
*dipA*_DR	ACGAATTCGAGCTCGGCTCTCGGCCGATGCCAT	
*gcbA*_UF	CGACGGCCAGTGCCACCAGCTTGGCGCGGGCCA	Amplify the upstream donor for constructing Δ*gcbA*
*gcbA*_UR	CCACGAAAGAAGCGCGTCTGGCCTTTTGTCATCAGC	

*gcbA*_DF	GCTGATGACAAAAGGCCAGACGCGCTTCTTTCGTGG	Amplify the downstream donor for constructing Δ*gcbA*
*gcbA*_DR	ACGAATTCGAGCTCGGTGGTGTAGCCGGTGACC	
*roeA*_UF	CGACGGCCAGTGCCATCGTAGCCCAGGCGGTTG	Amplify the upstream donor for constructing Δ*roeA*
*roeA*_UR	CATGCGGACCCGCAGAACCAATTGATCGGCCGGCCC	
*roeA*_DF	GGGCCGGCCGATCAATTGGTTCTGCGGGTCCGCATG	Amplify the downstream donor for constructing Δ*roeA*
*roeA*_DR	ACGAATTCGAGCTCGTTGCTCAACCGACGCCTG	
*morA*_UF	CGACGGCCAGTGCCAAACGGCACCGGGTTCGGG	Amplify the upstream donor for constructing Δ*morA*
*morA*_DF	CCCCAGGAATTCCGGTCAGCCGCCGTGCGGCGCGCT	
*morA*_DF	AGCGCGCCGCACGGCGGCTGACCGGAATTCCTGGGG	Amplify the downstream donor for constructing Δ*morA*
*morA*_DR	ACGAATTCGAGCTCGTCCCATGACGAGCGACTC	
*siaD*_UF	CGACGGCCAGTGCCATGGCCGACGAGGACGAGG	Amplify the upstream donor for constructing Δ*siaD*
*siaD*_UR	CAATCGCTGGAGCAACTGGCCTCCTCAGGCGTCCAG	
*siaD*_DF	CTGGACGCCTGAGGAGGCCAGTTGCTCCAGCGATTG	Amplify the downstream donor for constructing Δ*siaD*
*siaD*_DR	ACGAATTCGAGCTCGACCTGCACATACCCGGCA	
*fimX*_UF	CGACGGCCAGTGCCACCCTGCCCATCGTCAACC	Amplify the upstream donor for constructing Δ*fimX*
*fimX*_UR	GCCCAGGGCGCCCGTTCTGGAAAGGGCTCAGTCCGC	
*fimX*_DF	GCGGACTGAGCCCTTTCCAGAACGGGCGCCCTGGGC	Amplify the downstream donor for constructing Δ*fimX*
*fimX*_DR	ACGAATTCGAGCTCGCCGCGCCAGCTTCAAGGA	
*PA1851*_UF	CGACGGCCAGTGCCACTTCGGCACGCTAAAACC	Amplify the upstream donor for constructing Δ*PA1851*
*PA1851*_UR	CGAGGTAGGGGAGTCAGCAGGAAACGAAAAAGGCGC	
*PA1851*_DF	GCGCCTTTTTCGTTTCCTGCTGACTCCCCTACCTCG	

*PA1851*_DR	ACGAATTCGAGCTCGTCCCGAACACACCGAGCC	Amplify the downstream donor for constructing Δ*PA1851*
*PA2567*_UF	CGACGGCCAGTGCCACTCCTGCCCGCGCTAGAG	Amplify the upstream donor for constructing Δ*PA2567*
*PA2567*_UR	GTAGCCCAGGAGTCCAGCCCAATGCAGGCACGTGGA	
*PA2567*_DF	TCCACGTGCCTGCATTGGGCTGGACTCCTGGGCTAC	Amplify the downstream donor for constructing Δ*PA2567*
*PA2567*_DR	ACGAATTCGAGCTCGCGGCCATTGAGCGAAGCG	
*wspR*_UF	CGACGGCCAGTGCCAGCCAGCGAATCGAAGCTG	Amplify the upstream donor for constructing Δ*wspR*
*wspR*_UR	ATGGCCGCCGCGCCGCGTTTCTCTCCGGGACCGG	
*wspR*_DF	CCGGTCCCGGAGAGAAACGCGGCGCGGCGGCCAT	Amplify the downstream donor for constructing Δ*wspR*
*wspR*_DR	ACGAATTCGAGCTCGGTTCGGGTCCATCTCGCC	
*sadC*_UF	CGACGGCCAGTGCCACAACTCGCCCAGACGCAG	Amplify the upstream donor for constructing Δ*sadC*
*sadC*_UR	ACCGGGTGGTAGTAGGAGCATACGGGTCGGCGAGCG	
*sadC*_DF	CGCTCGCCGACCCGTATGCTCCTACTACCACCCGGT	Amplify the downstream donor for constructing Δ*sadC*
*sadC*_DR	ACGAATTCGAGCTCGGCCCATGGTCGGTACGGT	
*toxR*_UF	CGACGGCCAGTGCCATCGAGTCCTACCCAAGCC	Amplify the upstream donor for constructing Δ*toxR*
*toxR*_UR	TGGCAGCCGCGTCGGCGTAAGTGATGGCTCTATGGG	
*toxR*_DF	CCCATAGAGCCATCACTTACGCCGACGCGGCTGCCA	Amplify the downstream donor for constructing Δ*toxR*
*toxR*_DR	ACGAATTCGAGCTCGGATGGCTCCGTGGCCGAC	
*siaA*_UF	CGACGGCCAGTGCCACGGCCACCGCCCGGCAGT	Amplify the upstream donor for constructing Δ*siaA*
*siaA*_UR2	GTCGAGGAAACCGTCGGTGGCACAGGCCAGCAACAG	
*siaA*_DF2	CTGTTGCTGGCCTGTGCCACCGACGGTTTCCTCGAC	Amplify the downstream donor for constructing Δ*siaA*
*siaA*_DR	ACGAATTCGAGCTCGCAGCGATGCGGCCAGTGG	

*siaB*_UF	CGACGGCCAGTGCCACCGACCAGGCGATGCGCA	Amplify the upstream donor for constructing Δ*siaB*
*siaB*_UR2	GCGTAGCTGCTCCTTGTACAGGTCTAGCGTTTCCAT	
*siaB*_DF2	ATGGAAACGCTAGACCTGTACAAGGAGCAGCTACGC	Amplify the downstream donor for constructing Δ*siaB*
*siaB*_DR	ACGAATTCGAGCTCGGGCGTCCAGTTCGCGTTC	
*siaC*_UF	CGACGGCCAGTGCCAGAGGAGATAGGCCACGCG	Amplify the upstream donor for constructing Δ*siaC*
*siaC*_UR	GGGGAAGCTGCAGTCCTCGCCGGGTATGTGCAGGTC	
*siaC*_DF	GACCTGCACATACCCGGCGAGGACTGCAGCTTCCCC	Amplify the downstream donor for constructing Δ*siaC*
*siaC*_DR	ACGAATTCGAGCTCGCCCCAGGTATCGTTGACC	
*alg8*_UF	CGACGGCCAGTGCCATCCTGCCTGCCCAAGGAT	Amplify the upstream donor for constructing Δ*alg8*
*alg8*_UR	CGGTGGTCATACGATGGTGGCGAGGCCACGTTTGTA	
*alg8*_DF	TACAAACGTGGCCTCGCCACCATCGTATGACCACCG	Amplify the downstream donor for constructing Δ*alg8*
*alg8*_DR	ACGAATTCGAGCTCGATCGGCATGGGTGACGAA	
P*psl*_UF	CGACGGCCAGTGCCACGCCAATCCCGACAACCT	Amplify the upstream donor for constructing ΔP*psl*
P*psl*_UR	GTGGATAGGGAAGAGCAAGCCGCCAATCCGCGAGCG	
P*psl*_DF	CGCTCGCGGATTGGCGGCTTGCTCTTCCCTATCCAC	Amplify the downstream donor for constructing ΔP*psl*
P*psl*_DR	ACGAATTCGAGCTCGGTCCGGCACCAGCAGGAC	
*fabAB*_OF	cgtaatgaaattcaaCTGCAGTTCAGGGATTTT	Amplify *fabAB* from ATCC 27853
*fabAB*_OR	cctgcagagcactagTCAACCCTGCCAGCGCTT	
*wbpM*_UF	CGACGGCCAGTGCCAGCGATCCGTGCATGTGGA	Amplify the upstream donor for constructing PAO1 Δ*wbpM*
*wbpM*_UR	TTTCCGGAGAACGATGACCCTATTTATCAGCCCCGT	
*wbpM*_DF	ACGGGGCTGATAAATAGGGTCATCGTTCTCCGGAAA	

*wbpM*_DR	ACGAATTCGAGCTCGCCCGGTTCTTCTCCAGCA	Amplify the downstream donor for constructing PAO1 Δ*wbpM*
*siaC*_UF	cgacggccagtgccaGAGGAGATAGGCCACGCG	Amplify the upstream donor for constructing *siaC*^{T68A} and *siaC*^{T68D}
T68A_R	ACTGGCGTTCAGGTACAGCAGGCG	
T68D_R	AACTGTCGTTCAGGTACAGCAGGCG	
*siaC*_DR	acgaattcgagctcgCCCCAGGTATCGTTGACC	Amplify the downstream donor for constructing *siaC*^{T68A} and *siaC*^{T68D}
T68A_F	TGAACGCCAGTTCGATCAAGGCCA	
T68D_F	TGAACGACAGTTCGATCAAGGCCAT	
pJM_*siaA*_F2	aggaattcctcgagaGAGGCTACCTGCGTGGTC	Amplify P*sia-siaA* from ATCC 27853
pJM_*siaA*_R2	cggtatcgataagctGGTGCCCCTAGTCGAATC	
P*siaA*_UR2	tggcttactgaccatGGCTATCCCTATCAGTTT	Amplify the promoter region of *siaA* from ATCC 27853
*siaA*_IF2	ctgttggcggtacatCGCCTGCTGTTGCGCCCC	Amplify the sequence downstream of *siaA* TM2 from ATCC 27853
CusS_PF	ctgatagggatagccATGGTCAGTAAGCCATTT	Amplify the sequence encoding N-terminus to TM2 of CusS from *E. coli* MG1655
CusS_PR	gcgcaacagcaggcgATGTACCGCCAACAGTAC	
P*siaA*_F	cggtatcgataagctGCTGCTCCAGCAAGCCGC	Amplify P*sia* from ATCC 27853 and PAO1 for constructing P*sia-lux*
P*siaA*_R	aggaattcgatatcaGGCTATCCCTATCAGTTT	
*siaD*_OF	cgtaatgaaattcaaCCAGGCCCACGACGAGTA	Amplify *siaD* from ATCC 27853 for constructing P*rha-siaD*
*siaD*_OR	cctgcagagcactagGCCTGAGGAGGCTCAGCG	
*siaB*_F	cgtaatgaaattcaaCCTTCCGATTCGACTAGG	Amplify *siaB* from ATCC 27853 for constructing P*rha-siaB*
*siaB*_R	cctgcagagcactagTCAGATCACGGCGCGCAG	

P*psl*_F	cggtatcgataagctCGGCTAGCCACCTGGCCG	Amplify P*psl* from ATCC 27853 for constructing P*psl-lux*
P*psl*_R	aggaattcgatatcaGTTGTTTGCTCTGCCGAT	

Table S4. Differentially expressed PDE and DGC genes in ATCC 27853 following a temperature downshift from 37°C to RT identified by RNA-seq.

GGDEF domain

Locus tag		Gene	Log_2 fold change	Significance	Product
PAO1	ATCC 27853				
**PA0169**	**ACG06_00890**	***siaD***	1.56	yes	Diguanylate cyclase
PA0290	ACG06_01530	-	-1.58	no	Hypothetical protein
PA0338	ACG06_01785	-	0.32	no	Hypothetical protein
PA0847	ACG06_22585	-	-0.21	no	Diguanylate cyclase
**PA1107**	**ACG06_21250**	***roeA***	-1.72	no	RoeA
PA1120	ACG06_21175	*tpbB*	-0.72	no	Diguanylate cyclase TpbB
**PA1851**	**ACG06_17265**	**-**	-1.06	no	Hypothetical protein
PA2870	ACG06_10635	-	-0.33	no	Diguanylate cyclase
PA3177	ACG06_09075	-	-0.91	yes	Diguanylate cyclase
PA3343	ACG06_08200	*hsbD*	0.33	no	Diguanylate cyclase HsbD
**PA3702**	**ACG06_06150**	***wspR***	-0.4	no	WspR
**PA4332**	**ACG06_24555**	***sadC***	0.29	no	SadC
PA4396	ACG06_24885	-	0.63	no	Two-component response regulator
**PA4843**	**ACG06_27955**	***gcbA***	-0.77	yes	GcbA
PA4929	ACG06_28385	-	-0.48	no	Hypothetical protein

PA5487	ACG06_31295	-	-0.27	no	Hypothetical protein
PA2771	ACG06_11175	-	-0.84	no	Diguanylate cyclase with a self-blocked I-site, Dcsbis
PA3061	ACG06_09655	*pelD*	-0.45	no	PelD

GGDEF + EAL domains

Locus tag		Gene	Log_2 fold change	Significance	Product
PAO1	ATCC 27853				
PA0285	ACG06_01505	-	1.27	no	Conserved hypothetical protein
PA0575	ACG06_02995	*rmcA*	-0.48	no	Redox regulator of c-di-GMP, RmcA
**PA0861**	**ACG06_22515**	***rbdA***	-1.74	yes	RbDA
PA1181	ACG06_20865	-	-0.34	no	Conserved hypothetical protein
**PA1433**	**ACG06_20060**	**-**	-1.53	yes	Conserved hypothetical protein
PA1727	ACG06_17940	*muc*R	0.28	no	MucR
PA2072	ACG06_16125	-	-3.09	no	Conserved hypothetical protein
**PA2567**	**ACG06_13285**	**-**	0.64	yes	Hypothetical protein
PA3258	ACG06_08665	-	0.61	no	Hypothetical protein
PA3311	ACG06_08360	*nbdA*	-1.79	no	NbdA
PA4367	ACG06_24735	*bifA*	-0.6	no	BifA
**PA4601**	**ACG06_26550**	***morA***	-0.57	no	Motility regulator
**PA4959**	**ACG06_28545**	***fimX***	1.06	yes	FimX
**PA5017**	**ACG06_28845**	***dipA***	-1.55	yes	DipA
**PA5295**	**ACG06_30300**	**-**	1.36	yes	Hypothetical protein
PA5442	ACG06_31070	-	0.25	no	Conserved hypothetical protein

EAL domain

Locus tag		Gene	Log_2 fold change	Significance	Product
PAO1	ATCC 27853				
PA2133	ACG06_15850	-	inf	no	Cyclic-guanylate-specific phosphodiesterase
PA2200	ACG06_15525	-	-0.85	no	Cyclic-guanylate-specific phosphodiesterase
PA3825	ACG06_05480	-	-0.55	no	Diguanylate phosphodiesterase
**PA3947**	**ACG06_04885**	***rocR***	0.84	no	RocR
**PA0707**	**ACG06_24070**	***toxR***	-2.56	no	Transcriptional regulator ToxR
PA2818	ACG06_10910	*arr*	1.27	yes	Aminoglycoside response regulator

HD-GYP domain

Locus tag		Gene	Log_2 fold change	Significance	Product
PAO1	ATCC 27853				
**PA2572**	**ACG06_13255**	**-**	-3.17	yes	Probable two-component response regulator
**PA4108**	**ACG06_04105**	**-**	-2.67	yes	Cyclic di-GMP phosphodiesterase
**PA4781**	**ACG06_27635**	**-**	-2.73	yes	Cyclic di-GMP phosphodiesterase

Table S5. A list of DGC- and PDE-encoding genes subjected to gene deletion analysis.

Locus tag		Gene	Domain signature*	Product
PAO1	ATCC 27853			
PA0169	ACG06_00890	*siaD*	GGEEF	Diguanylate_cyclase
PA1107	ACG06_21250	*roeA*	GGEEF	RoeA
PA1851	ACG06_17265	-	GGEEF	Hypothetical protein
PA3702	ACG06_06150	*wspR*	GGEEF	WspR
PA4332	ACG06_24555	*sadC*	GGEEF	SadC
PA4843	ACG06_27955	*gcbA*	GGEEF	GcbA
PA0861	ACG06_22515	*rbdA*	GGDEF, ELL	RbDA
PA1433	ACG06_20060	-	RGGEF, KVL	Conserved hypothetical protein
PA2567	ACG06_13285	-	SPTFR, EAL	Hypothetical protein
PA4601	ACG06_26550	*morA*	GGDEF, EAL	motility regulator
PA4959	ACG06_28545	*fimX*	GDSIF, EVL	FimX
PA5017	ACG06_28845	*dipA*	ASNEF, EAL	DipA
PA5295	ACG06_30300	-	GSDEF, EAL	Hypothetical protein
PA3947	ACG06_04885	*rocR*	EVL	RocR
PA0707	ACG06_24070	*toxR*	EAL	Transcriptional regulator ToxR
PA2572	ACG06_13255	-	YN-GYP	Probable two-component response regulator
PA4108	ACG06_04105	-	HD-GYP	Cyclic di-GMP phosphodiesterase
PA4781	ACG06_27635	-	HD-GYP	Cyclic di-GMP phosphodiesterase

*: as illustrated by Valentini et al. (6).

Table S6. Differentially expressed fatty acids biosynthesis and degradation genes in ATCC 27853 following a temperature downshift from 37°C to RT identified by RNA-seq

Locus tag		Log2fold change	Gene	Product
PAO1	ATCC 27853			
PA4848	ACG06_27980	1.14	*accC*	biotin carboxylase
PA2968	ACG06_10130	1.09	*fabD*	malonyl-CoA-[acyl-carrier-protein] transacylase
PA2965	ACG06_10145	1.55	*fabF1*	beta-ketoacyl-acyl carrier protein synthase II
PA2967	ACG06_10135	1.39	*fabG*	3-oxoacyl-[acyl-carrier-protein] reductase
PA3645	ACG06_06740	1.21	*fabZ*	(3R)-hydroxymyristoyl-[acyl carrier protein] dehydratase
PA3299	ACG06_08430	2.53	*fadD1*	long-chain-fatty-acid--CoA ligase
PA3300	ACG06_08425	-1.41	*fadD2*	long-chain-fatty-acid--CoA ligase

Data S1. RNA-seq data.

Data S2. Lipidomics data.

Supplementary References

1. Kumagai, Y., Yoshizawa, S., Nakamura, K., Ogura, Y., Hayashi, T., andKogure, K. (2017) Complete and draft genome sequences of eight oceanic Pseudomonas aeruginosa strains Genome Announc 5, e01255-01217,

2. Choi, K.-H., andSchweizer, H. P. (2005) An improved method for rapid generation of unmarked Pseudomonas aeruginosa deletion mutants BMC Microbiol 5, 30-30 10.1186/1471-2180-5-30

3. Hoang, T. T., Karkhoff-Schweizer, R. R., Kutchma, A. J., andSchweizer, H. P. (1998) A broad-host-range Flp-FRT recombination system for site-specific excision of chromosomally-located DNA sequences: application for isolation of unmarked Pseudomonas aeruginosa mutants Gene 212, 77-86,

4. Meisner, J., andGoldberg, J. B. (2016) The Escherichia coli rhaSR-PrhaBAD inducible promoter system allows tightly controlled gene expression over a wide range in Pseudomonas aeruginosa Appl Environ Microbiol 82, 6715-6727,

5. Becher, A., andSchweizer, H. P. (2000) Integration-proficient Pseudomonas aeruginosa vectors for isolation of single-copy chromosomal lacZ and lux gene fusions BioTechniques 29, 948-952 10.2144/00295bm04

6. Valentini, M., andFilloux, A. (2016) Biofilms and Cyclic di-GMP (c-di-GMP) Signaling: Lessons from Pseudomonas aeruginosa and Other Bacteria J Biol Chem 291, 12547-12555 10.1074/jbc.R115.711507
